# Sustainability of Diets in Mexico: Diet Quality, Environmental Footprint, Diet Cost, and Sociodemographic Factors

**DOI:** 10.3389/fnut.2022.855793

**Published:** 2022-05-27

**Authors:** Katherine Curi-Quinto, Mishel Unar-Munguía, Sonia Rodríguez-Ramírez, Juan A. Rivera, Jessica Fanzo, Walter Willett, Elin Röös

**Affiliations:** ^1^Center for Research on Nutrition and Health, National Institute of Public Health, Cuernavaca, Mexico; ^2^National Institute of Public Health, Cuernavaca, Mexico; ^3^Nitze School of Advanced International Studies, Bloomberg School of Public Health, Johns Hopkins University, Baltimore, MD, United States; ^4^Department of Nutrition, Harvard School of Public Health, Boston, MA, United States; ^5^Department of Energy and Technology, Swedish University of Agricultural Sciences, Uppsala, Sweden

**Keywords:** sustainable diet, Mexico, diet cost, environmental footprint, carbon footprint, land use, water footprint

## Abstract

**Background:**

Little is known about the current intake of sustainable diets globally and specifically in middle-income countries, considering nutritional, environmental and economic factors.

**Objective:**

To assess and characterize the sustainability of Mexican diets and their association with sociodemographic factors.

**Design:**

Dietary data of 2,438 adults within the National Health and Nutrition Survey 2012 by integrating diet quality measured by the Healthy Eating Index (HEI-2015), diet cost, and four environmental indicators were analyzed: land use (LU), biodiversity loss (BDL), carbon footprint (CFP), and blue water footprint (BWFP). We defined healthier more sustainable diets (MSD) as those with HEI-2015 above the overall median, and diet cost and environmental indicators below the median. Logistic regression was used to evaluate the association of sociodemographic factors with MSD.

**Results:**

MSD were consumed by 10.2% of adults (4% of urban and 22% of rural), who had lower intake of animal-source foods, unhealthy foods (refined grains, added sugar and fats, mixed processed dishes and sweetened beverages), fruits, and vegetables, and higher intake of whole grains than non-MSD subjects. Characteristics of MSD vs. non-MSD (urban; rural) were: HEI-2015 (62.6 vs. 51.9; 66.8 vs. 57.6), diet-cost (1.9 vs. 2.8; 1.9 vs. 2.5 USD), LU (3.3 vs. 6.6; 3.2 vs. 5.9 m^2^), BDL (105 vs. 780; 87 vs. 586 species × 10^−10^), BWFP (244 vs. 403; 244 vs. 391 L), and CFP (1.6 vs. 4.4; 1.6 vs. 3.7 kg CO_2_eq). Adults from rural vs. urban (OR 2.7; 95% CI: 1.7, 4.1), or from the South (OR 2.1; 95% CI: 1.1, 3.9), Center (OR 2.3; 95% CI: 1.3, 4.4) vs. the North were more likely to consume MSD, while adults with high vs. low socioeconomic status were less likely (OR 0.17; 95% CI: 0.09, 0.3).

**Conclusions:**

The MSD is a realistic diet pattern mainly found in disadvantaged populations, but diet quality is still sub-optimal. Increased consumption of legumes, fruits, and vegetables, and a reduction in unhealthy foods, is required to improve nutritional quality of diets while ensuring their environmental sustainability.

## Introduction

Globally, there is a growing need to promote not only healthy and affordable diets, but also more environmentally sustainable diets (1–3). Food systems currently contributes one-third of global greenhouse gas emissions (GHGE) (2), uses 50% of available land, and is responsible for up to 70% of freshwater use (1, 2, 4).

In Mexico, food production is a major user of freshwater resources and land, with land use for agriculture being the main driver of deforestation and biodiversity loss (5, 6). The Mexican food system contributes one-third of national GHGE (7). In the past 20 years, consumption of animal-source foods and processed foods high in energy, sodium, added sugar, and saturated fats and low in nutrients, e.g., sweetened beverages and sweet and salty snacks (8), has increased and consumption of whole grain and legumes has decreased. Consistent with economic theory (9), the proportion of spending on food decreased as income increased (Engel's law). Specifically, the share for basic foods such as legumes and some cereals decreased (Bennet's law), while the share on animal source food, fruits and vegetables and non-basic products increased in the wealthier households (10). This is contributing to an epidemic of obesity and non-communicable chronic diseases, coexisting with nutritional deficiencies (11). Thus, there is a growing need to transform Mexico's food system and promote healthy and sustainable diets.

Sustainable diets, defined by the Food and Agriculture Organization (FAO) as those that “*are protective and respectful of biodiversity and ecosystems, culturally acceptable, accessible, economically fair and affordable; nutritionally adequate, safe and healthy; while optimizing natural and human resources*” (12), comprise different dimensions (nutritional, economic, environmental, cultural), and there is no standardized way to analyze the sustainability of diets (13, 14). Most studies on sustainable diets derive from high-income settings, and most limit their analysis and interpretation to the nutritional dimension and one or a few environmental indicators (mainly GHGE) (15–19). Few studies have analyzed the sociodemographic factors associated with sustainable diets (20–23), which are important when tailoring policy recommendations to a local context.

Sustainable diets have different environmental footprints and costs in low- and middle-income countries compared with high-income countries (24–27). Studies to date show that diets with lower consumption of animal-source foods generally reduce the carbon footprint and land use (1, 28–31). However, some diets high in fruit and vegetables have a relatively high water footprint (32). More sustainable and healthier diets can be more expensive in lower-income compared with higher-income countries (25, 27), but a modeling study in Mexico, which is considered an upper-middle income country by the World Bank, showed that sustainable diets could be achieved at a lower cost than the average diet (33). Dietary patterns in Mexico also differ with sociodemographic characteristics (34–36). This variability, and the current lack of data on the environmental footprint of Mexican diets, emphasize the need to assess the sustainability of Mexican adult diets and their association with sociodemographic factors. Therefore, the aim of this study is to assess and characterize the sustainability of Mexican diets, using indicators of diet quality, diet cost, and environmental footprints; and to analyze the association between the consumption of relatively more sustainable diets with sociodemographic factors. To our knowledge, this is the first study to integrate all these aspects when analyzing the Mexican diet.

## Methods

### Study Design and Population

The study sample was obtained from the National Health and Nutrition Survey (ENSANUT-2012), a stratified and multi-stage random dietary survey conducted between October 2011 and May 2012 with representativeness at national, state, and rural/urban level (37). Although the ENSANUT-2012 is not representative at the municipal level, it has information on the municipality and locality in which each individual interviewed lives, which allows food price data to be linked at the municipal level from other income and expenditure survey, as explained in the diet cost assessment.

From an initial sample of 2,792 adults (18- to 59-year-olds), we excluded 147 pregnant or lactating women and 207 adults with implausible nutrient intake. Therefore, our analytical sample was 2,438 adults with complete dietary and sociodemographic data (see the flow chart in Supplementary Figure 1). The dietary data derived from responses to a semi-quantitative 7-day food frequency questionnaire (SFFQ), collected by trained interviewers using standardized methodology (37). The SFFQ was validated with a 24-h recall and included 140 foods items classified into 14 groups that contributed more than 90% of total energy and nutrient intake (38). The study protocol for the survey was approved by the Ethics Committee of the National Institute of Public Health in Mexico (INSP).

### Overview of the Diet Sustainability Assessment

Based on FAO definition of sustainable diets (12) and the methodological approach used by Masset et al. (20), we assessed the sustainability of the diets by integrating the nutritional, environmental and economic components using indicators of diet quality (using the HEI-2015), diet cost, and four environmental impact indicators (land use, biodiversity loss, water and carbon footprint). We used the median values of each indicator for the overall population as cutoff points to identify adults consuming a healthier and more sustainable diet (MSD). We defined the MSD as having higher diet quality (HEI-2015 above the overall median value), lower diet cost, and lower environmental footprint (below the overall median value). We established a comparison group considering those adults whose diet did not meet these three conditions (non-MSD). We compared the dietary characteristics of adults consuming a MSD with those who do not have a more sustainable diet (non-MSD), and we assessed the association between the consumption of MSD with sociodemographic factors. As part of the description, we also presented the characteristics of the average diet (corresponding to the overall study sample), and diets with high-quality (above the median), low cost and low-environmental impact (below the median). The schematic overview of the diet sustainability assessment is presented in Supplementary Figure 2.

#### Assessment of Indicators of Diet Sustainability

##### Diet Quality Assessment

Diet quality was assessed using the Healthy Eating Index (HEI-2015), a validated method for assessing overall diet quality in adults according to the American Dietary Guidelines (39). HEI-2015 is based on analysis of food groups and nutrients grouped into nine adequacy components (recommended for a healthy diet): total fruits, whole fruits, total vegetables, greens and beans, whole grains, dairy, total protein foods, seafood and plant proteins, and fatty acids (ratio of poly- and mono-unsaturated to saturated fatty acids); and four moderation components (to be limited in a healthy diet): refined grains, sodium, added sugars, and saturated fats. Each component can contribute from 0 to 5 or 0 to 10 points, so the total score ranges from 0 to 100. To calculate the HEI-2015 for individuals, we followed the procedures described in detail on the National Cancer Institute website (40). In the case of added sugar, we followed the methodology of Louie et al. (41). We adapted the original food grouping to avoid double counting in the diet cost and environmental footprint analyses. For the “greens and beans” component, we included only beans and other legumes, for “total protein foods” we included only animal-food protein (no seafood), and for “seafood and plant proteins” we included seafood, seeds, and nuts (see food groups and scoring in Supplementary Table 1).

##### Diet Cost Assessment

Daily diet cost per person was estimated by adding up the product of the quantity consumed for each food (as reported in the SFFQ) and its average unit price at the municipality level as described below. To compare the dietary characteristics between individuals, the daily diet cost was adjusted to 2000 kcal, which is close to the average daily energy intake in adults (>19 years) in Mexico (1,958 kcal/day) as reported in a previous study based on SFFQ 2012 (42).

We obtained data on food prices at municipality level from the 2012 National Survey of Household Income and Expenditure (ENIGH), which applied a stratified probabilistic design with national representativeness for urban and rural areas (43). We estimated food prices by dividing the total monetary expenditure by the quantity purchased by households in the previous week and obtained median prices by municipality. For milk in the national program “*Liconsa”*, we used its subsidized price for 2012 (44). When prices at municipality level were missing, we used the median values of food prices at state level or in urban/rural areas. To reduce potential measurement error, we excluded food items with quantities and prices in the 1st or 99th percentile of the distribution. We replaced prices above two standard deviations with the average price for each food item plus two standard deviations (45). All prices were adjusted for inflation to the year 2018 using the National Consumer Price Index (46) and converted into dollars (USD) using the average exchange rate (19.23 Mexican pesos per USD) (47), the latter to allow comparability with international studies. We matched food prices to the SFFQ food items manually, and then linked them to each person in ENSANUT-2012 according to their geographical residence. For municipalities in ENSANUT-2012 for which prices were lacking in ENIGH, we assigned the prices in the nearest municipality based on geographical location coordinates provided by INEGI-2010, using the Stata module “*Geonear*” (48).

##### Environmental Footprint Assessment

The environmental footprint of diets was assessed using indicators for land use (LU), biodiversity loss (BDL), carbon footprint (CFP), and blue water footprint (BWFP). We estimated each indicator per kg of food item in the SFFQ (as described in detail below), then multiplied this by the amount of food consumed per person. Land use, BDL, and BWFP were estimated for primary production, while CFP was estimated from cradle to distribution center. We considered food losses during post-harvest, handling and storage, processing, distribution, and consumption as estimated by FAO (49).

The methodological scheme, a further explanation of the indicators used, and all detailed data used to estimate the environmental indicators of the food in the SFFQ are presented in Supplementary Material (Section Methods; Supplementary Figures 3, 4; Supplementary Tables 2–10).

#### Land Use

Use of agricultural land, defined as the area of land needed to produce one kg food (m^2^/kg), was estimated for plant-based foods by dividing the amount of the crop needed to obtain one kg of raw food by the average country-specific yield (kg/m^2^) obtained in the period 2008–2012 according to the FAOSTAT database (50). We accounted for the contribution of land from imported foods by estimating the weighted average land use based on the contribution of the importing country to the total food supply in Mexico. We used data from National Mexican Agriculture Planning (2017–2030) (51) and the Statistical Yearbook of Foreign Trade of Mexico (2008–2012) (52) to identify imported foods, countries of origin, and their contribution. We estimated the land required to produce 1 kg of animal-source food based on animal feeding requirements. We followed the same steps as for plant-based food to estimate the land use for each component of the animal feed ration, then aggregated the values to obtain the land required per kg of food for animal feed. Since the feed ration composition differs with animal species and with production system, we accounted for the contribution of each system to the total production of each species. Considering these factors, we calculated the land use per kg of final product from cattle, chicken, and pigs. For cattle, we included two product orientations (pure meat production and dairy systems) and two production systems (grazing and mixed). For chicken, we considered two orientations (pure meat broilers and eggs), and three production systems (meat broiler, egg backyard, and egg layer). For pigs, we included three production systems (backyard, intermediate, and industrial). Country-specific data on these orientation and production systems were taken from the Global Livestock Environmental Assessment Model (GLEAM) report issued by FAO. Data on feed ration composition by production system for Mexico were obtained from the interactive GLEAM-i tool, where estimates are made using a modeling framework based on life cycle assessment (LCA) that simulates the activities and processes involved in livestock production (53). GLEAM operates at (sub) national, regional, and global scale. Detailed data on the feed ration composition and the parameters for animal production are presented in Supplementary Tables 4–10.

#### Biodiversity Loss

Biodiversity loss, expressed as the average number of potential species lost × 10^10^ per kg of food, representing the biodiversity damage caused by land occupation for food production, was estimated using the methodology of Chaudhary et al. (54). In brief, we obtained characterization factors representing potential species loss for mammals, birds, amphibians, reptiles, and plants from food production occupying 1 m^2^ of land, according to the type of land use (cropland for all plant-based foods, pasture for ryegrass and alfalfa) and production intensity based on land management intensity (low, light, or intense). We calculated the biodiversity loss for intense land use based on monoculture farming with no crop rotation, use of inorganic fertilizer, and use of an irrigation system (55). We multiplied the factor for the land needed to produce one kg of food by the amount of each food consumed according to the SFFQ.

#### Blue Water Footprint

Blue water footprint, expressed as L per kg of food, was calculated as the total amount of blue water (groundwater and surface water) needed to produce 1 kg of food, taken from Mekonnen and Hoekstra (56). The estimates obtained corresponded to the average water consumption 1996–2005 for different crops, livestock, and derived products for Mexico. In a similar manner as for the land use estimates, we accounted for the contribution of the BWFP of imported foods. This indicator measures consumptive water use, but does not capture the extent to which this water use is problematic in the region where the food is produced (57), which is a limitation. Lack of data on where within a country (primarily Mexico and the US in this case) the food commodities are grown prevented such an assessment (see Section Methods in Supplementary Material for more details).

#### Carbon Footprint

Carbon footprint was calculated as kg of carbon dioxide equivalents (kg CO_2_e/kg) using the metric Global Warming Potential over 100 years (GWP_100_) (58) (see Supplementary Material for more on this indicator and its limitations). Data on the CFP for plant-based food items were obtained from a systematic review of existing LCA studies by Clune et al. (59). Global averages were used, since CFP values specific for Mexico were not available. The CFP estimates for plant-based foods included the total GHGE from primary production to distribution center. In the case of animal-source food items, which have considerably higher CFP values, country-specific CFP values were obtained from the GLEAM-I estimates for Mexico (60).

### Sociodemographic Variables

Sociodemographic variables considered were: sex (male/female), age, and socioeconomic status (SES) (all included in tertiles), education level, categorized as low (elementary school or no education), medium (high school), or high (university), ethnicity (indigenous and non-indigenous), and place of residence (area and region). We based SES on an index of household wellbeing constructed by ENSANUT using component analysis of household characteristics, goods, and services (61). We categorized ethnicity following ENSANUT's methodology as indigenous for an individual speaking any indigenous language, or otherwise non-indigenous. We defined area of residence as rural (locations with <2,500 inhabitants) or urban (locations with ≥2,500 inhabitants), and divided region of residence into North, Center, Mexico City, and South.

### Statistical Analysis

We calculated the mean diet characteristics of the average diet, the individual components of diet sustainability (high-quality diet, low-cost diet, low environmental footprint diet), MSD and non-MSD by urban and rural, and determined the relative percentage difference of each type of diet in comparison with the average diet. We assessed differences between dietary characteristics and composition of diet by food groups for adults with MSD and non-MSD, using the *t*-test. We used multivariate logistic regression to evaluate the association between sociodemographic characteristics and consumption of MSD (yes/no). As part of the characterization of diet sustainability, we explored the association between each environmental indicator of the diet (LU, BDL, CFP, BWFP) and diet quality (HEI-2015 score), using the Spearman correlation coefficient (rho). We stratified all the analyses by urban and rural area, since descriptive analysis showed different patterns for all indicators. We performed sensitivity analysis on the definition of MSD by including only the nutritional and environmental dimensions, and excluding the diet cost. For the analyses, we used the Stata software version 14.0 and the complex survey module (SVY) to consider the probabilistic design of the survey and expansion factors, and considered a *p*-value < 0.05 to be statistically significant.

## Results

On average, the overall sample had daily energy intake of 1,898 kcal/day (95% CI: 1,854, 1,941), a HEI-2015 value of 54.1 points (95% CI: 53.5, 54.7), and a daily diet cost of 48.9 MXN (95% CI: 47.6, 50.2) or 2.5 USD (95% CI: 2.5, 2.6). The per capita daily environmental footprint for LU, BDL, CFP, and BWFP was 5.8 m^2^ (95% CI: 5.6, 6.0), 657 × 10^−10^ species potentially lost (95% CI: 610, 703); 3.8 kg CO_2_e (95% CI: 3.7, 4.0), and 357 L (95% CI: 347, 367), respectively.

**Figure 1:**
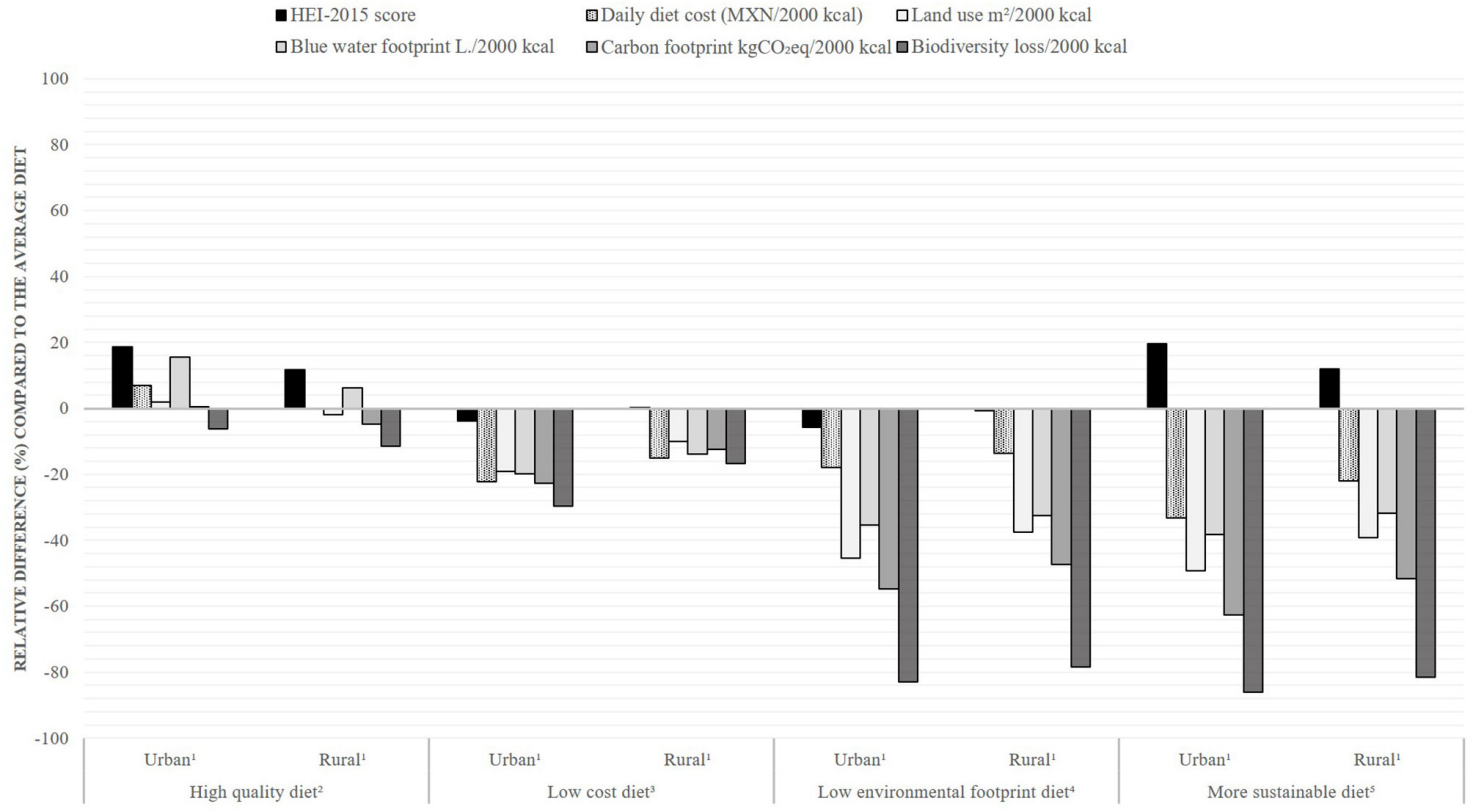
Difference in dietary indicators of high-quality, low-cost, low-environmental footprint, and more sustainable diets relative to the average diet, by area of residence in Mexico. Values presented are the percentage difference in each dietary indicator (HEI-2015, cost and environmental footprint) relative to the average diet. ^1^Urban: more than 2,500 inhab; Rural: less than 2,500 inhab. ^2^Diets with HEI-2015 score above the overall median (54.2). ^3^Diets with daily diet cost below the median (50.9 MXN ≈ 2.6 USD). ^4^Diets with all environmental indicators below the median: land use (5.5 m^2^), blue water footprint (361 L), carbon footprint (3.4 kgCO_2_eq), and biodiversity loss (423 potential species loss × 10^−10^). ^5^Diets combining the criteria for high-quality, low-cost, and low-environmental-footprint diets. HEI-2015, Healthy Eating Index.

Table 1 displays the dietary characteristics of the average diet, diets with high quality, low cost, low environmental footprint, and MSD and non-MSD by area of residence. In comparison with the average urban diet, the average rural diet had significantly lower energy intake, lower cost, higher HEI-2015, and lower environmental footprint (*p* < 0.05 for all indicators). According to our definition of MSD, 10.2% of adults; 4.1% (95% CI: 3.1, 5.4%) of the urban population and 22.4% (95% CI: 18.7, 26.5%) of the rural population, consumed MSD. In terms of the relative difference (%) compared with the average diet, the high-quality diet had a higher cost in urban areas (+7%) with no extra cost in rural areas, and higher BWFP (+16% and +6%) and lower BDL (−6% and −11%) in urban and rural areas, respectively. The high-quality diet had lower CFP than the average diet (−5%) only in rural areas (Figure 1). The low-cost diet had lower diet quality in urban areas (−4%) and no quality difference in rural areas, and had a lower environmental footprint (range −10 to −30%) than the average diet. The low-environmental footprint diet had a lower cost both in urban and rural areas (−18% and −14%) but also lower diet quality (−6% and −1%). As expected, MSD had a higher HEI-2015 value (+20% in urban and +12% in rural) and lower diet cost and environmental footprint than the average diet (Figure 1).

**Table 1:** Characteristics of the average diet and of high-quality, low-cost, low-environmental footprint, and more sustainable diets (MSD), by area of residence in Mexico (*n* = 2,438).

	**Average diet^**a**^**	**High-quality diet^**b**^**	**Low-cost diet^**c**^**	**Low-environmental footprint diet^**d**^**	**More sustainable diet (MSD)^**e**^**	**Non-sustainable** **diet (non-MSD)^**f**^**	**MSD vs.** **non-MSD**
	**Mean** **(95% CI)^**g**^**	**Mean** **(95% CI)^**g**^**	**Mean** **(95% CI)^**g**^**	**Mean** **(95% CI)^**g**^**	**Mean** **(95% CI)^**g**^**	**Mean** **(95% CI)^**g**^**	***p*-value**
**Sample size**							
Urban *n* (%)^f^	1,636 (77.0)	725 (41.0)	681 (43.8)	364 (21.8)	84 (4.1)	1,512 (93.4)	-
Rural*n* (%)^f^	802 (23.0)	494 (65.6)	538 (69.7)	300 (38.8)	165 (22.4)	637 (88.5)	-
**Daily energy intake (kcal)**							
Urban	1,926 (1,872, 1,981)	1,887 (1,809, 1,965)	1,951 (1,876, 2,027)	1,909 (1,802, 2,015)	1,963 (1,750, 2,177)	1,925 (1,869, 1,981)	0.215
Rural	1,804 (1,749, 1,859)^*^	1,787 (1,717, 1,857)	1,831 (1,764, 1,898)	1,861 (1,773, 1,949)	1,840 (1,731, 1,949)	1,793 (1,729, 1,858)	0.47
**HEI-2015 score**							
Urban	52.4 (51.7, 53.1)	62.1 (61.6, 62.7)	50.4 (49.3, 51.4)	49.4 (48.0, 50.7)	62.6 (60.9, 64.3)	51.9 (51.2, 52.7)	<0.001
Rural	59.7 (58.6, 60.8)^*^	66.7 (65.9, 67.6)	59.8 (58.4, 61.2)	59.3 (57.5, 61.1)	66.8 (65.5, 68.0)	57.6 (56.4, 58.9)	<0.001
**Daily diet cost (MXN/2,000 kcal)**							
Urban	54.0 (52.8, 55.2)	57.7 (56.1, 59.4)	41.9 (41.3, 42.6)	44.4 (42.6, 46.2)	36.1 (33.9, 38.2)	54.8 (53.6, 56.0)	<0.001
Rural	45.8 (44.3, 47.3)^*^	45.8 (44.0, 47.6)	38.9 (38.00, 39.78)	39.6 (37.6, 41.6)	35.7 (34.4, 37.1)	48.7 (47.0, 50.5)	<0.001
**Daily diet cost (USD/2,000 kcal)**							
Urban	2.8 (2.7, 2.9)	3.0 (2.9, 3.1)	2.2 (2.1, 2.2)	2.3 (2.2, 2.4)	1.9 (1.8, 2.0)	2.8 (2.8, 2.9)	<0.001
Rural	2.4 (2.3, 2.5)^*^	2.4 (2.3, 2.5)	2.0 (2.0, 2.1)	2.1 (1.95, 2.16)	1.9 (1.8, 1.9)	2.5 (2.4, 2.6)	<0.001
**Land use (m** ^**2**^ **/2,000 kcal)**							
Urban	6.5 (6.2, 6.7)	6.6 (6.3, 6.9)	5.2 (5.0, 5.5)	3.5 (3.4, 3.6)	3.3 (3.1, 3.5)	6.6 (6.4, 6.8)	<0.001
Rural	5.3 (344, 376)^*^	5.2 (4.9, 5.5)	4.8 (4.5, 5.0)	3.3 (3.2, 3.5)	3.2(3.1, 3.4)	5.9 (5.6, 6.2)	<0.001
**Blue water footprint L./2,000 kcal**							
Urban	396 (384, 409)	458 (439, 477)	318 (305, 330)	256(247, 265)	244 (223, 265)	403 (390, 415)	<0.001
Rural	358 (342, 374)^*^	380 (359, 402)	309 (295, 323)	241 (230, 253)	244 (230, 258)	391 (372, 410)	<0.001
**Carbon footprint kgCO2eq/2,000 kcal**							
Urban	4.3 (4.1, 4.5)	4.3(4.1, 4.6)	3.3 (3.1, 3.5)	2.0 (1.9, 2.0)	1.6 (1.5, 1.8)	4.4 (4.3, 4.6)	<0.001
Rural	3.2 (3.0, 3.4)^*^	3.1 (2.8, 3.3)	2.8 (2.6, 3.1)	1.7 (1.59, 1.81)	1.6 (1.4, 1.7)	3.7 (3.5, 4.0)	<0.001
**Potential species loss per/2,000 kcal**							
Urban	752 (696.2, 807)	705 (636, 775)	529 (470, 588)	129 (111, 147)	105 (77.6, 133)	780 (722, 838)	<0.001
Rural	474 (414, 535)^*^	420 (352, 489)	395 (323, 468)	102 (80.1, 124.8)	87.4 (64.0, 110.8)	586 (511, 661)	<0.001

a *Overall mean.*

b *Diets with HEI-2015 above the overall median of the population (54.2).*

c *Diets with cost below the overall median (50.9 MXN ≈ 2.6 USD).*

d *Diets with environmental indicators below the overall median: land use (5.5 m^2^), blue water footprint (361 L), carbon footprint (3.4 kgCO2eq), and biodiversity loss (423 potential species loss × 10^–10^).*

e *Diets that combine the criteria for high-quality, low-cost and low-environmental-footprint diets. All the groups (2–5) are non-independent.*

f *Diets that do not meet the criteria for high-quality, low-cost and low-environmental-footprint diets.*

g *Percentage and mean values are adjusted by the probabilistic survey design.*

* *Indicates significant difference compared with the average diet in urban area (p < 0.05)*.

Comparison of the food group composition of diets revealed that both in urban and rural areas, adults following a MSD had higher intake of whole grains and lower intake of animal-source food (except eggs), added sugar, non-sweetened drinks, fruits, and vegetables than those not following a MSD. In urban areas only, intake of sweetened drinks and seafood & nuts was lower in MSD than non-MSD, while in rural areas intake of refined grains was lower in MSD than in non-MSD. We found no statistically significant differences in intake of legumes, eggs, added fats, and mixed processed dishes between MSD and non-MSD (Table 2).

**Table 2:** Comparison of intake of food groups among adults with and without more sustainable diets (MSD) in urban and rural areas of Mexico.

	**URBAN**			**RURAL**		
	**Non-MSD^**a**^ (*n* = 1,512)**	**MSD^**b**^ (*n* = 84)**	*p*-value*	**Non- MSD^**a**^ (*n* = 637)**	**MSD^**b**^ (*n* = 165)**	*p*-value*
**Composition of diet by food groups (g)**	**Mean (95% CI)**	**Mean (95% CI)**		**Mean (95% CI)**	**Mean (95% CI)**	
Whole fruits	209 (193, 226)	123 (103, 142)	<0.001	235 (211, 258)	124 (105.5, 142)	<0.001
Vegetables	197 (187, 207)	150 (123, 176)	0.001	190 (173, 207)	147 (130, 163)	<0.001
Legumes	18.6 (17.0, 20.2)	24 (17.4, 30.1)	0.088	28.5 (25.3, 31.8)	27.0 (22.2, 31.9)	0.61
Whole-grain foods	45.6 (40.6, 50.6)	251 (190, 312)	<0.001	132 (113, 150)	403 (358, 448)	<0.001
Seafood and nuts	8.9 (7.8, 9.9)	4.9 (2.2, 7.7)	0.008	6.5 (5.0, 8.1)	8.2 (4.9, 11.5)	0.351
Dairy	245 (229, 261)	124 (83.5, 164)	<0.001	215 (193, 237)	108 (78.3, 137)	<0.001
Beef	30.1 (27.6, 32.5)	2.7 (1.5, 3.8)	<0.001	22.4 (19.2, 25.6)	2.0 (1.0, 3.0)	<0.001
Poultry	21.6 (19.4, 23.7)	14.0 (7.6, 20.5)	0.021	19.6 (16.5, 22.7)	8.7 (6.28, 11.20)	<0.001
Eggs	33.8 (31.1, 36.6)	44.9 (33.6, 56.3)	0.087	36.5 (32.9, 40.2)	35.7 (28.9, 42.4)	0.827
Pork	34.0 (31.4, 36.6)	16.7 (12.1, 21.2)	0.001	26.4 (23.5, 29.2)	16.6 (11.29, 22.0)	<0.001
Refined-grain foods	245 (235, 255)	229 (175, 282)	0.567	195 (179, 210)	101.8 (77.7, 125.9)	<0.001
Added sugars	43.9 (40.9, 47.0)	20.3 (13.2, 27.4)	<0.001	40.6 (36.1, 45.0)	26.6 (21.6, 31.6)	<0.001
Added fats	11.6 (11.0, 12.3)	10.8 (7.8, 13.7)	0.573	11.6 (10.3, 12.8)	9.0 (6.53, 11.44)	0.06
Mixed processed dishes	52.1 (47.0, 57.1)	40.5 (28.2, 52.9)	0.098	50.7 (42.3, 59.0)	44.3 (27.0, 61.5)	0.514
Sweetened drinks	288 (265, 311)	183 (121, 244)	0.002	217 (190, 244)	184 (149.1, 220)	0.169
Non-sweetened drinks	154 (135, 173)	83.7 (36.8, 131)	0.004	136 (113, 159)	86.2 (57.4, 115)	0.009

a *Adults without MSD.*

b *Adults with MSD consisting of diets with HEI-2015 above the median, and diet cost and environmental indicators (land use, biodiversity loss, blue water and carbon footprint) below the median of the overall diet. The mean values presented were estimated considering the complex design of the Mexican National Health and Nutrition Survey (ENSANUT 2012).*

* *The significance was assessed at p < 0.05 using a t-test for mean comparison with survey data. MSD, more sustainable diets; HEI-2015, Healthy Eating Index*.

**Figure 2:**
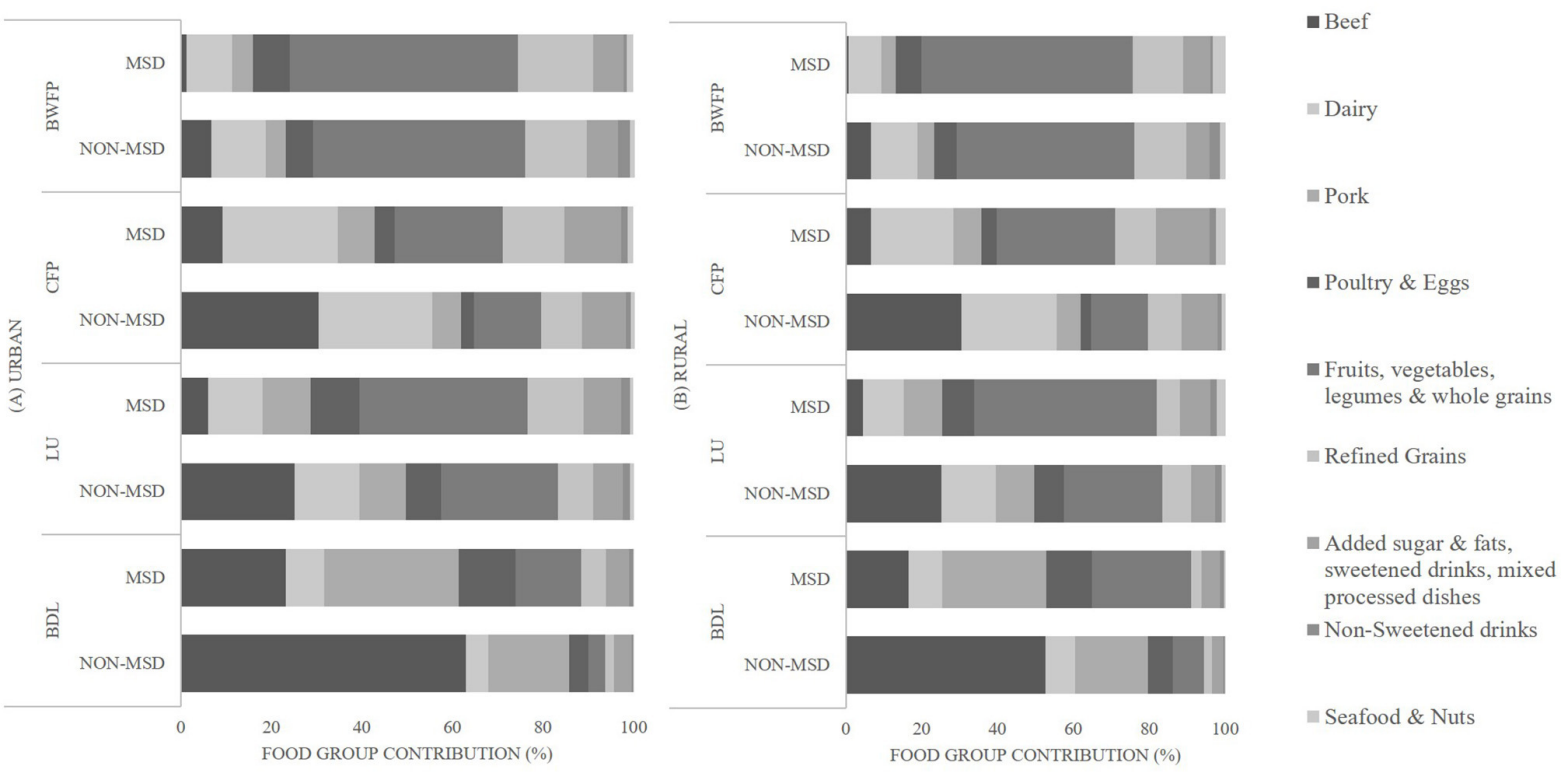
Contribution (%) of food groups to the total environmental footprint indicators among adults with MSD and non-MSD in **(A)** urban and **(B)** rural areas of Mexico. BDL, Biodiversity loss (potential number of species lost/2,000 kcal × 10^−10^); LU, land use (m^2^/2,000 kcal); CFP, carbon footprint (kgCO_2_eq/2,000 kcal); BWFP, blue water footprint (L/2,000 kcal); MSD, more sustainable diet (diets with HEI-2015 above the median, diet cost and environmental indicators (land use, biodiversity loss, blue water and carbon footprint) below the median of the overall diet.

The relative contribution of food groups to each environmental footprint indicator differed between adults according to type of diet (Figure 2). In both urban and rural areas, for those not following a MSD, beef, dairy, and pork were the major contributors to BDL, LU and CFP, while plant-based food groups (fruit and vegetables, legumes, whole grains) were the major contributor to BWFP. Refined grains and the group of added sugar and fats, sweetened drinks, and mixed processed dishes were the second largest contributors to CFP. For those following a MSD, the contribution of beef, dairy, and pork for all environmental footprint indicators was lower than for those not following a MSD, and the contribution of plant-based food groups, poultry and eggs was higher (Figure 2). For all diets in urban and rural areas, non-sweetened beverages and seafood and nuts made the lowest contributions to the environmental footprint indicators.

There were indications of an association between sociodemographic factors and MSD (Table 3). Adults from rural areas were 2.7 times more likely to follow a MSD than adults from urban areas, while adults from the South and the Center were more likely to consume a MSD than adults from the North (odds ratio (OR) 2.3 and 2.1, respectively). On the other hand, adults with medium and high SES were less likely to consume a MSD than adults with low SES (OR: 0.46 and 0.17, respectively). Age, sex, education, and ethnicity were not associated with consumption of a MSD (Table 3).

**Table 3:** Sociodemographic characteristics associated with consumption of more sustainable diets (MSD) in Mexico.

**More sustainable diet**	**OR^**a**^ Adjusted (95% CI)**	***p*-value**
**Age group (tertiles)**		
18.0–<29.6	Reference	
29.6–<43.5	0.94 (0.58, 1.52)	0.801
43.5–59.0	0.88 (0.55, 1.40)	0.582
**Gender**		
Male	Reference	
Female	0.86 (0.59, 1.25)	0.418
**Education level** ^**b**^		
Low	Reference	
Medium	1.54 (0.70, 3.36)	0.281
High	0.57 (0.22, 1.48)	0.249
**Socioeconomic level** ^**c**^		
Low	Reference	
Medium	0.46 (0.29, 0.74)	0.001
High	0.17 (0.09, 0.32)	<0.001
**Ethnicity** ^**d**^		
Indigenous	Reference	
Non-indigenous	0.96 (0.53, 1.72)	0.880
**Area of residence (%)**		
Urban	Reference	
Rural	2.67 (1.73, 4.13)	<0.001
**Region of residence (%)**		
North	Reference	
Center	2.12 (1.14, 3.94)	0.017
Mexico City	0.37 (0.08, 1.69)	0.198
South	2.34 (1.26, 4.37)	0.007

a *Estimated Odds Ratio coefficient from logistic regression model (n = 2,438) considering the complex design of the Mexican National Health and Nutrition Survey (ENSANUT 2012).*

b *Categorized as low (elementary school or no education), medium (high school), or high (university).*

c *Based on an index of household wellbeing constructed by ENSANUT using principal component analysis of household characteristics, goods, and services.*

d *Based on language spoken, categorized as indigenous (when the adult spoke any indigenous language) or non-indigenous*.

Regarding the association between diet quality and the environmental indicators, we found that HEI-2015 score had a positive association with BWFP (rho 0.3; *p* < 0.001), but an inverse association with CFP (rho −0.09; *p* < 0.001), and BDL (rho −0.14; *p* < 0.001) (Supplementary Table 11).

Sensitivity analysis using the definition of MSD excluding the diet cost showed similar, but slightly lower, values for diet quality, mainly in urban areas, and similar values for the environmental footprint indicators, than the original definition (Supplementary Figure 3).

## Discussion

As far as we know, this is one of the first studies among middle-income countries and in the Latin American region to assess the environmental footprint of food and to link them with dietary data to characterize nutritional, economic, and environmental dimensions of diet sustainability using a National Survey with individual dietary data. We found that in Mexico, a small proportion of adults consume a healthier and more sustainable diet (10.2% nationally; 4% of urban and 22% of rural area). The MSD is a realistic diet pattern mainly found in disadvantaged populations but diet-quality is still sub-optimal and requires improvements.

Mexican adults following MSD had lower intake of animal-source food groups (mainly beef, dairy, pork), higher intake of plant-based food (mainly legumes and whole grains) (62, 63), lower intake of fruits and vegetables (undesirable), and lower intake of unhealthy food groups (refined grains, added sugar and fats, processed mixed dishes and sweetened beverages). This confirms that MSD can be achieved by increasing plant-based foods and decreasing animal-source and unhealthy foods, to improve diet quality while decreasing environmental footprint and diet costs (1, 23, 28). Consumption of fruits and vegetables in MSD should be promoted, while considering strategies to avoid increased diet costs.

Having lower SES and living in rural, South, or Central Mexico (which have the highest rates of poverty in Mexico) (64), were positively associated with having a healthier and more sustainable dietary pattern. A study on apparent food consumption (per-capita food availability) in Mexico showed that the richest consumed more animal-source foods, oils, and sugars, representing higher energy intake, with 60–80% higher land requirements than the diets of the poorest (58, 64). Other studies have found that adults with high SES in Mexico have less sustainable diets than indigenous or rural-dwelling adults (23). A recent study that analyzed the same survey showed that a higher SES was negatively associated with the quality of diet in urban and rural areas, and that a high-quality diet was more expensive in urban but not in rural areas at all SES levels (65). Nutrient-dense food such as fruits, animal and dairy products, but also some ultra-processed products are income elastic (66), which partly explains that lower-income groups have relatively more sustainable diets, since they consume less of these foods. This suggests that economic constraints as well as sociocultural and geographical factors are associated with MSD consumption, confirming previous findings of a negative relationship of SES with diet quality and a positive relationship with environmental footprint, due to higher consumption of animal-source and unhealthy products (34, 67, 68). In addition, the differences in food consumption patterns among place of residence are associated with a heterogeneous process of nutrition transition that was consistently found in previous reports in Mexico (33, 34, 63). Also, there are differences in food supply among areas and regions that determine food access and prices and quality (69), for instance, people from rural areas have more access to home produced food as these are the main places of food production in Mexico, while in the wealthier region of the North, supermarkets are the main supply of food and in the South and Center the open market is used more (11, 69, 70).

There is no standardized method for identification of MSD. Some define MSD using a theoretical reference diet, whereas we identified MSD relative to the average diet in the study population. Most studies define MSD with only indicators of nutritional adequacy and one environmental footprint indicator (22, 31, 63, 71), while we included diet quality, four environmental footprint indicators, and diet cost (13, 20, 57). The MSD characteristics identified were consistent with those found for other populations (31, 64, 71). Characteristics of MSD in the Mexican population were closer to the EAT Lancet Commission Healthy Reference Diet (EAT-HRD) (28) than the average diet for added fats and sugars and whole grains (mainly in rural areas), while beef and pork intake was below/close to EAT-HRD. However, intake of legumes, fruits, vegetables, seafood, nuts, and dairy were below the EAT-HRD. Similarly, the environmental footprint of MSD was close to the absolute planetary boundaries for CFP and LU (1.9 kg CO_2_e and 5.0 m^2^ per capita and day, respectively) (72).

Sensitivity analysis of MSD excluding diet costs showed that high-quality diets with low environmental footprint cost less than the average diet, as found in a recent modeling study on food baskets in Mexico (33). Although healthy food costs more than non-healthy food in Mexico (73) and diet cost is positively associated with diet quality in urban areas, a higher-quality diet can be achieved at similar cost as a lower-quality diet, since cost distributions overlap (65). The lower cost of MSD compared with average diets in Mexico could be explained by reduced consumption of unhealthy products and animal-source food such as red meat, which cost more than whole grains and legumes present in higher amounts in MSD. We did not assess the affordability of MSD, due to lack of reliable information regarding income in ENSANUT, but we compared the average cost of MSD in our sample with the extreme poverty line in the country in 2018 (1.8 USD in rural areas, 2.5 USD in urban areas) (74). We found that MSD could be more affordable than the average diet, since in urban areas it cost 24% less than the extreme poverty line and in rural areas only 5% more, whereas the average diet cost exceeded the extreme poverty line by 12 and 25% in urban and rural areas, respectively.

Although MSD as defined had relatively better quality than the average diet, we highlight that it still had a sub-optimal diet quality score (62 and 69 points in urban and rural areas, respectively, compared with an optimum of >80 points) (36). This diet lacks sufficient fruits and vegetables, and still presents consumption of some unhealthy products such as sweetened beverages, processed dishes and ultra-processed products, confirming the need to improve the quality of the diet of Mexican adults (36) to meet their nutritional requirements in addition to being sustainable. This is relevant especially in rural areas, where the lower environmental footprint of MSD was associated with lower intake of animal-source foods, higher intake of whole grains and legumes, and lower intake of fruit and vegetables. This lower intake of certain food groups could reduce the diversity of their diets resulting in potential micronutrient deficiency (75). In a context of food insecurity, such as it happens in several communities in Mexico, consuming regional and seasonal fruits and vegetables can reduce problems of availability and high price of this food groups. Additionally, strategies for promoting sustainable diets among these vulnerable populations should support consumption of modest amounts of low environmental impact animal-source foods, such as poultry, eggs and pig, and increase access to fruits, vegetables, legumes and nuts. Furthermore, improvements in diet quality could be achieved without increasing its total cost by selecting healthy and sustainable food options (33, 65).

This study has some limitations. The CFP for plant-based food was derived from an international meta-analysis (59), which did not represent food production systems in Mexico or account for different levels of processing (76). For animal-source foods, CFP and LU were estimated using the GLEAM-I tool, which has major uncertainties in feed use especially for ruminants. Not accounting for different types of land (e.g., pasture vs. cropland) penalizes diets high in ruminant products, as ruminants can produce food on less productive land (see Section Methods in Supplementary Material for other details). Despite these uncertainties we have used the most systematized and update data to estimate the environmental footprint of Mexican diets, also as those errors are systematic, we do not expect any differential bias. Regarding health, the limited list of foods in the SFFQ could have underestimated food intake, as could underreporting (e.g., unhealthy foods) because of social desirability (77). However, SFFQ is a validated method and the foods contributed more than 90% of total energy and nutrient intake (38). The analyzed survey is 10 years old, but another study showed that HEI-2015 as diet quality indicator has not changed between ENSANUT 2006, 2012, and 2016 (35), and differences on dietary intake by place of residence are similar to those found in ENSANUT 2018-19 (78). Although HEI-2015 includes food groups and nutrients, processed foods had to be disaggregated into added sugar, sodium, fats, etc. to assess diet quality. Hence, HEI-2015 only captures the role of energy-dense and nutrient-poor foods in the diet through their ingredients. It can also generate errors in estimation of vitamin/mineral retention during cooking. However, HEI-2015 has been used for comparison between groups of people in relation to sustainability (79).

Among the strengths, this is the first study in Mexico and one of the first studies in middle-income countries and Latin America to assess the environmental footprint for +130 commonly consumed foods, based on a systematized dataset on primary production, which could be used by other similar countries in the region to link them with diet information or adapt the methodology to estimate its own environmental footprint indicators, instead of using information from high-income countries. We analyzed a representative national survey that used standardized methods to reduce potential selection bias and measurement errors, and we were the first to link indicators of diet quality, diet cost, and environmental footprint to measure diet sustainability considering nutritional, economic and environmental dimensions. This approach including the environmental impact of food production linked with food consumption opens a new line of analysis and platform of discussion in the field of public health and population nutrition. This may help to generate recommendations not only for healthy but also for more environmentally sustainable diets that are in line with the current sustainable development goals.

Our results highlight the urgent need to promote sustainable diets that incorporate high-quality diets at lower environmental footprints and accessible cost, considering the differences in food patterns by SES and area of residence. For this, our study provides methods and environmental footprint estimates of foods and diets for the formulation of sustainable food-based dietary guidelines for Mexico that are currently being updated and for the first time will consider an environmental approach, and our estimations of food environmental footprints could also be used by similar countries to include environmental sustainability indicators into their dietary guidelines. Also, further studies that complement our analysis of diet quality considering the nutrients requirements for different age groups will be useful to promote a more sustainable and healthy diet for all of the population. We also highlight the need to improve/refine estimates of the environmental impact of processed foods, as limited data were found regarding their ingredients and methods of processing and packaging to estimate the carbon footprint of food production for Mexico. Also, data were not available for estimation of environmental impact indicators considering the different regional and local food production systems, for example, the comparison of more traditional vs. modern systems. Similarly, further analysis is needed of drivers of local food consumption and production systems by place of residence; this information can be useful for designing comprehensive policies to promote/maintain MSD considering socioeconomic, cultural, and geographical heterogeneities. This is particularly relevant for rural, South and Central regions of Mexico, which consistently showed better diets than average, but still not optimal diets.

In conclusion, this study provides estimation of the environmental footprint of most frequently consumed food in Mexico and a systematic methodology that could be used by other middle-income countries to assess diet sustainability considering nutritional, economic and environmental aspects. Among the sociodemographic factors associated to relatively MSD diets we found that compared with the average diet, a small proportion of Mexican adults in urban areas, and almost one-fifth in rural areas, had MSD characterized by lower intake of animal-source foods and unhealthy foods, and higher intake of whole grains, although intake of fruits and vegetables was low. Diets are relatively healthier and more sustainable among low vs. high income settings, but its nutritional quality is still suboptimal and there is need to further improve sustainable diets in Mexico through adding more legumes, fruits, and vegetables and reducing unhealthy products. Improving the economic conditions of the population will lead less sustainable diets, so promoting diets that are nutritionally adequate, affordable diets with a low environmental footprint is necessary to ensure the health of the planet and the population.

## Data Availability Statement

The original contributions presented in the study are included in the article/Supplementary Material, further inquiries can be directed to the corresponding author/s.

## Ethics Statement

The studies involving human participants were reviewed and approved by the Ethics Committee of the National Institute of Public Health in Mexico (INSP). The patients/participants provided their written informed consent to participate in this study.

## Author Contributions

KC-Q designed the study, conducted the data analysis, and wrote the manuscript. ER and MU-M assisted with the design, data analysis, interpretation, and drafting the manuscript. SR-R assisted with the data analysis and revision of the manuscript. JR, WW, and JF contributed to interpretation of the results and the discussion, and assisted in drafting the manuscript. KC-Q and MU-M had primary responsibility for the final content. All authors have read and approved the final manuscript.

## Funding

This is part of a research-based doctoral thesis by KC-Q, who received a PhD scholarship from the Mexican National Council of Science and Technology (Grant Number: 797149/616402). The funder had no role in the study design, analyses, or interpretation.

## Conflict of Interest

The authors declare that the research was conducted in the absence of any commercial or financial relationships that could be construed as a potential conflict of interest.

## Publisher's Note

All claims expressed in this article are solely those of the authors and do not necessarily represent those of their affiliated organizations, or those of the publisher, the editors and the reviewers. Any product that may be evaluated in this article, or claim that may be made by its manufacturer, is not guaranteed or endorsed by the publisher.

## Abbreviations

BDL: Biodiversity loss
CFP: Carbon footprint
DGA: Dietary Guidelines for Americans
EAT-HRD: EAT-Lancet Healthy Diet Recommendation
ENIGH: National Household Income and Expenditure Survey
ENSANUT: National Health and Nutrition Survey
FAO: Food and Agriculture Organization
GLEAM: Global Livestock Environmental Assessment Model
GHGE: Greenhouse gas emissions
HEI-2015: Healthy Eating Index 2015
INSP: National Institute of Public Health in Mexico
INEGI: National Institute of Statistic, Geography and Informatics
LU: Land use
LCA: Life cycle assessment
MSD: Healthier and more sustainable diets
MXN: Mexican pesos
SFFQ: Semi-quantitative food frequency questionnaire
BWFP: Blue water footprint.
