# Supplementary material for: Sustainability of Diets in Mexico: Diet Quality, Environmental Footprint, Diet Cost, and Sociodemographic Factors
Source: Front Nutr. 2022 May 27;9:855793. doi: 10.3389/fnut.2022.855793 (PMC9185856; doi:10.3389/fnut.2022.855793)
Supplement: Supplementary file 1 [file Data_Sheet_1.pdf]

Supplementary Material

Title: Sustainability of diets in Mexico: diet quality, environmental footprint, diet cost, and sociodemographic factors

Authors:

Authors names: Katherine Curi-Quinto[1], Mishel Unar-Munguía[1], Sonia Rodríguez-Ramírez[1], Juan A. Rivera[2], Jessica Fanzo[3], Walter Willett[4], Elin Röös[5]

[1]Center for Research on Health and Nutrition, National Institute of Public Health, Cuernavaca, Morelos, Mexico. kcuri@iin.sld.pe

[2]National Institute of Public Health, Cuernavaca, Morelos, México.

[3]Nitze School of Advanced International Studies, Bloomberg School of Public Health, Johns Hopkins University, Baltimore, MD USA

[4]Department of Nutrition, Harvard School of Public Health, Boston, MA, USA.

[5]Department of Energy and Technology, Swedish University of Agricultural Sciences, Uppsala, Sweden.

Table of Contents

Supplementary Figure 1. Flow chart of the study sample. 4

Supplementary Figure 2. Overview of the diet sustainability assessment. 5

Supplementary Table 1. Dietary components and scoring criteria used for construction of HEI-2015 6

Supplementary Figure 3. Results of sensitivity analysis using the definition of more sustainable diets but excluding the diet cost. 8

Supplementary methods: Environmental footprint indicators....9

Supplementary Figure 4. Methodological scheme for estimation of environmental footprint indicators of plant-based food using dietary data from SFFQ 11

Supplementary Figure 5 Methodological scheme for estimation of environmental footprint indicators of animal-based food using dietary data from SFFQ 12

Supplementary Table 2. Data used to calculate the environmental footprint indicators of plant-based food in SFFQ-ENSANUT 2012....13

Supplementary Table 2 (Continued)**.** Data used to calculate the environmental footprint indicators of plant-based food in SFFQ-ENSANUT 2012 14

Supplementary Table 2 (Continued)**.** Data used to calculate the environmental footprint indicators of plant-based food in SFFQ-ENSANUT 2012 15

Supplementary Table 2 (Continued)**.** Data used to calculate the environmental footprint indicators of plant-based food in SFFQ-ENSANUT 2012 16

Supplementary Table 2 (Continued)**.** Data used to calculate the environmental footprint indicators of animal-based food in SFFQ-ENSANUT 2012 17

Supplementary Table 3. Data used to calculate the environmental footprint indicators of processed animal and plant-based food in SFFQ-ENSANUT 2012....18

Supplementary Table 3 (Continued)**.** Data used to calculate the environmental footprint indicators of processed animal and plant-food in SFFQ-ENSANUT 2012....19

Supplementary Table 4. Feed ration composition (%) for meat and dairy by production system, based on GLEAM-i data for Mexico 20

Supplementary Table 5. Average feed ratio composition (%) for meat and dairy by production system, based on GLEAM-i data for Mexico 22

Supplementary Table 6. Feed ratio composition (%) for chicken and pig by production system, based on GLEAM-i data for Mexico 24

Supplementary Table 7. Parameters for cattle production used in estimation of environmental footprint indicators by orientation and production system, based on GLEAM-i data for Mexico......25

Supplementary Table 8. Parameters for chicken and pig production used in estimation of environmental footprint indicators by production system, based on GLEAM-i data for Mexico.......26

Supplementary Table 9. Average land use and average potential species loss per kg dry matter (DM) of each animal feed ration component 27

Supplementary Table 10. Land use, biodiversity loss, and parameters used to estimate the carbon footprint of animal-based food in SFFQ. 29

Supplementary Table 11. Spearman correlation coefficients between diet quality (HEI-2015) and different environmental footprint indicators 31

Supplementary Figure 1. Flow chart of the study sample.

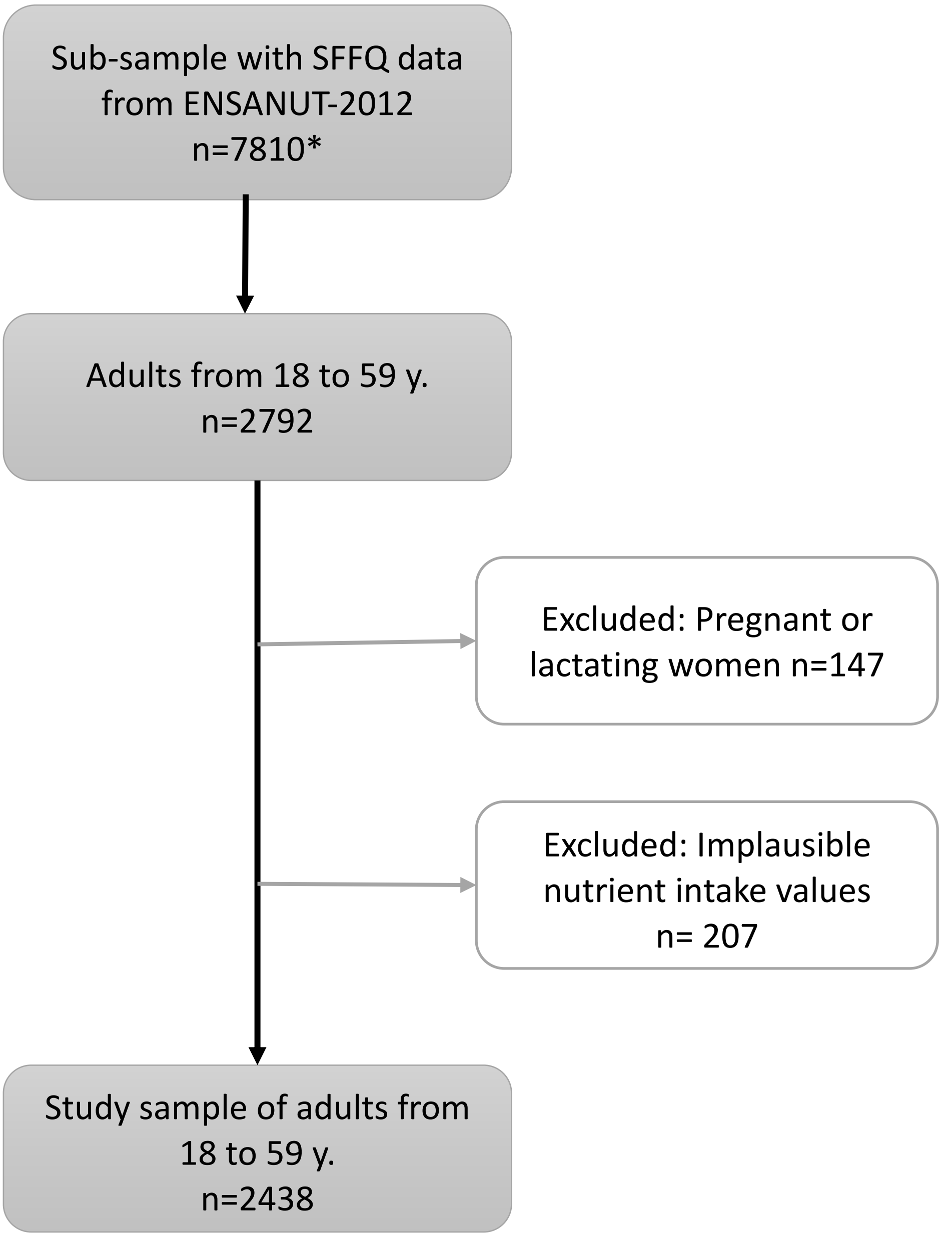

*Includes population from different age groups (from pre-school children to older adults).

Based on Ramirez et al. (2016). Methodology for estimating dietary data from the semi-quantitative food frequency questionnaire (SFFQ) of the Mexican National Health and Nutrition Survey 2012 (ENSANUT-2012).

Supplementary Figure 2. Overview of the diet sustainability assessment.

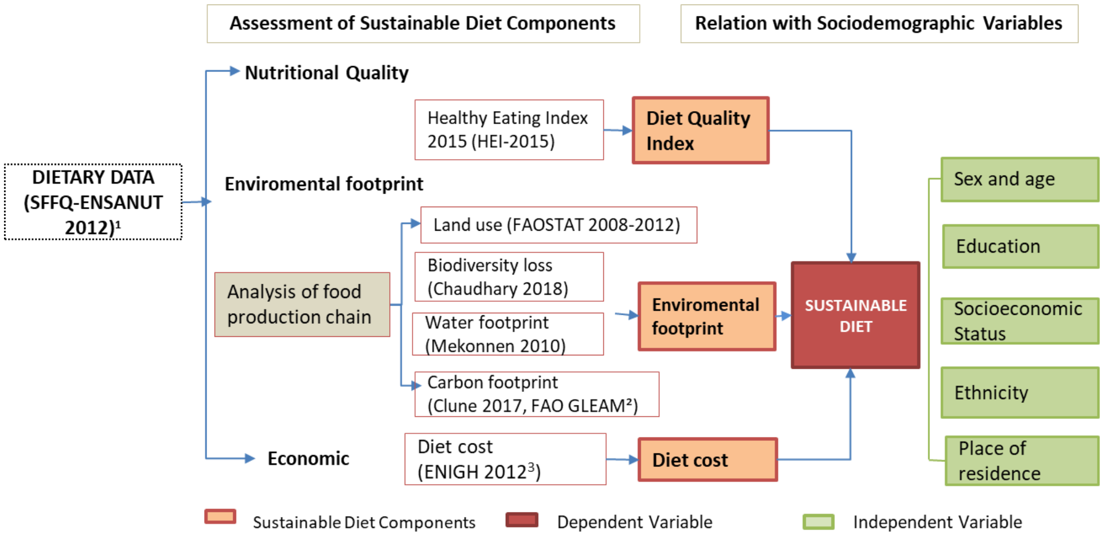

[1]National Health and Nutrition Survey. [2]Global Livestock Environmental Assessment Model [3]National Income and Expenditures Survey

Supplementary Table 1. Dietary components and scoring criteria used for construction of HEI-2015

Dietary component	Maximum	Standard for maximum	Standard for minimum
**Adequacy:**			
Total Fruits[2]	5	≥0.8 cup equiv. per 1,000 kcal	No fruit
Whole Fruits[3]	5	≥0.4 cup equiv. per 1,000 kcal	No whole fruit
Total Vegetables[4*]	5	≥1.1 cup equiv. per 1,000 kcal	No vegetables
Greens and Beans[5*]	5	≥0.2 cup equiv. per 1,000 kcal	No dark green vegetables or legumes
Whole Grains	10	≥1.5 oz equiv. per 1,000 kcal	No whole grains
Dairy[6]	10	≥1.3 cup equiv. per 1,000 kcal	No dairy
Total Protein Foods[7*]	5	≥2.5 oz equiv. per 1,000 kcal	No protein foods
Seafood & Plant Proteins[8*]	5	≥0.8 oz equiv. per 1,000 kcal	No seafood or plant proteins
Fatty Acids[9]	10	(PUFAs + MUFAs)/SFAs ≥2.5	(PUFAs + MUFAs)/SFAs ≤1.2
**Moderation:**			
Refined Grains	10	≤1.8 oz equiv. per 1,000 kcal	≥4.3 oz equiv. per 1,000 kcal
Sodium	10	≤1.1 gram per 1,000 kcal	≥2.0 grams per 1,000 kcal
Added Sugars	10	≤6.5% of energy	≥26% of energy
Saturated Fats	10	≤8% of energy	≥16% of energy

*The units for each dietary component are called unit equivalents, for example the unit equivalent for total fruits is the cup equivalent per 1,000 kcal, for whole grains it is the ounce equivalent per 1,000 kcal, etc.

[1]Intakes between the minimum and maximum standards are scored proportionately.
[2]Includes 100% fruit juice.
[3]Includes all forms except juice.
[4*]Originally included legumes (beans and peas). In this study we included only vegetables and fresh green peas.
[5*]Originally included legumes (beans and peas). In this study we included only legumes (beans, lentils, chickpeas, etc.)
[6]Includes all milk products, such as fluid milk, yogurt, and cheese, and fortified soy beverages.
[7*]Originally included legumes (beans and peas). In this study we included only protein from animal sources.
[8*]Originally included seafood, nuts, seeds, soy products (other than beverages), and legumes (beans and peas). In this study we excluded soy products and legumes.
[9]Ratio of poly- and monounsaturated fatty acids (PUFAs and MUFAs) to saturated fatty acids (SFAs).

Based on NCI. Developing the Healthy Eating index. Available from https://epi.grants.cancer.gov/hei/developing.html

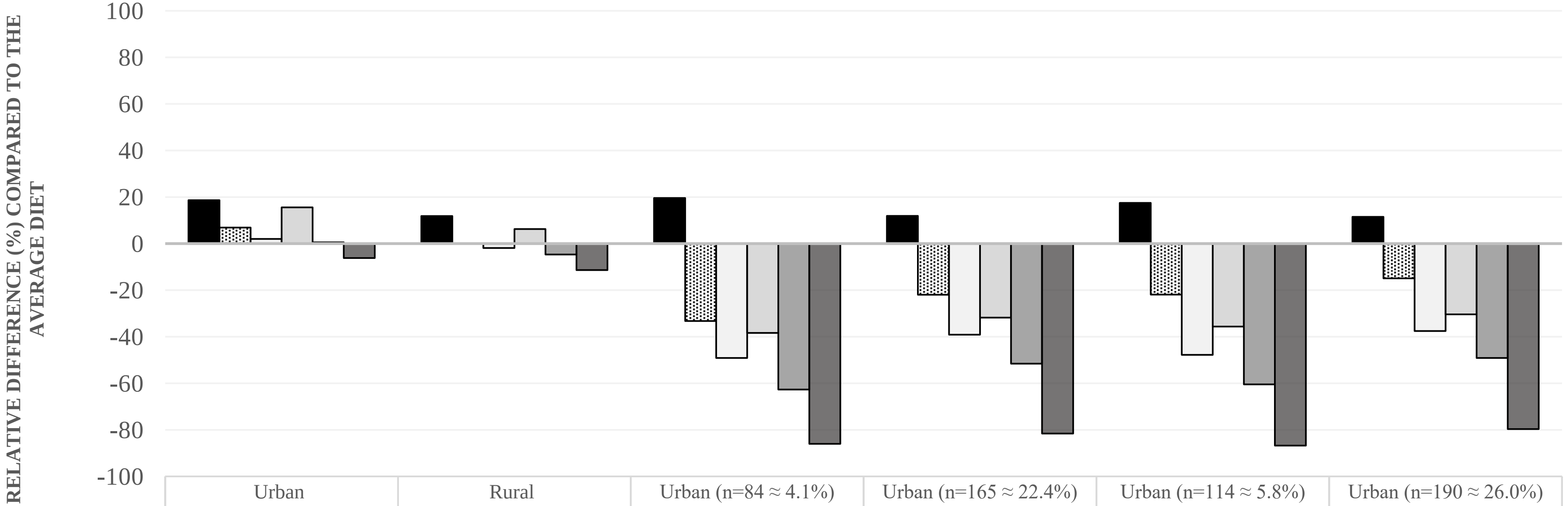

	Urban	Rural	Urban (n=84 ≈ 4.1%)	Urban (n=165 ≈ 22.4%)	Urban (n=114 ≈ 5.8%)	Urban (n=190 ≈ 26.0%)
	High quality diet [2]		More sustainable diet [4]		More sustainable diet [5]	
HEI-2015 score	19	12	20	12	17	11
Daily diet cost (MXN/2000 kcal)	7	0	-33	-22	-22	-15
Land use m²/2000 kcal	2	-2	-49	-39	-48	-38
Blue water footprint L./2000 kcal	16	6	-38	-32	-36	-30
Carbon footprint kgCO_2eq/2000 kcal	1	-5	-63	-52	-60	-49
Biodiversity loss/2000 kcal	-6	-11	-86	-82	-87	-80

Supplementary Figure 3. Results of sensitivity analysis using the definition of more sustainable diets but excluding the diet cost. Relative difference (%) compared to average diet of HEI-2015 score, daily diet cost, and environmental footprint indicators (land use, blue water footprint, carbon footprint, and biodiversity loss), among the high-quality diets, and two definitions of more sustainable diets compared to the habitual diet by area of residence. Diets with HEI-2015 score above the median of the HEI-2015 of their respective reference group. [5]Diets that combine the criteria for high-quality, low-cost and low-environmental footprint diets. [6]Diets that combine the criteria for high-quality and low-environmental footprint diets.

Supplementary methods: Environmental footprint indicators

To assess the environmental sustainability of the Mexican diets, we selected a set of commonly used environmental indicators, or environmental footprints (1). Below we justify the choice of indicators and briefly describe their limitations.

For estimating the contribution to climate change from the different diets, we used the carbon footprint (CFP) of foods, defined as estimated total amount of GHG emitted from a life cycle perspective from the product under study (2). CFP is an environmental indicator that shows emissions of greenhouse gases (GHG) including carbon dioxide (CO_2), methane (CH_4), and nitrous oxide (N_2O) to the atmosphere. There are different metrics available to aggregate the different GHGs into one metric (3), here we used the commonly applied Global Warming Potential (GWP) (4). However, GWP has several limitations, e.g., it requires an arbitrary choice of time horizon, which heavily affects the results and underrepresents longer-term climate impacts. Therefore several alternative metrics have been suggested (3) and alternative approaches for aggregating emissions from agriculture to better represent how emissions affect temperature change are being discussed (5). However, all metrics have limitations and their suitability ultimately depends on the application (3,6). In this study, we used GWP over a 100-year perspective (GWP_{100}) for the following reasons: 1) It is the most commonly used carbon footprint metric to date, thus enabling comparisons with other studies, 2) despite its limitations, it provides a valuable measure of the climate impact averaged over the coming hundred years, and 3) it is the metric for which data are available. The GLEAM-I estimates for animal-source food in Mexico use GWP factors from the AR5 IPCC report (7) (methane: 34, nitrous oxide: 298), while most of the studies estimating global average CFPs in the review by Clune et al. (8) probably used factors from the previous AR4 IPCC report (9) (methane: 25, nitrous oxide: 298) for plant-based foods. Thus, there is a difference in the methane factor used for animal-source foods and plant-based foods. However, rice is the only plant-based food associated with substantial methane emissions. Rice is not a staple in the Mexican diet to the same extent as maize and wheat (average daily intake of rice is 8.8 g per person, compared with 22.7 g tortilla and 23.1 g white bread). Using the lower methane factor for rice involved an underestimation of the CFP of the average Mexican diet of less than 0.5%, indicating that this inconsistency had no major impact on the results.

The blue water footprint (BWFP) as defined by Hoekstra et al. (2011)(10), was used to estimate the amount of water needed to produce the Mexican diets. BWFP is defined as *"Volume of surface and groundwater consumed as a result of the production of a product. Consumption refers to the volume of freshwater used and then evaporated or incorporated into a product. It also includes water abstracted from surface or groundwater in a catchment and returned to another catchment or the sea. It is the amount of water abstracted from groundwater or surface water that does not return to the catchment from which it was withdrawn"*. To assess the impact on water resources associated with consumptive blue water use, the water scarcity in the region where different crops are grown needs to be considered (11). There are several methods to account for water scarcity (12,13). For example, the AWARE method (14) supplies region- and country-specific water scarcity factors that can be multiplied by consumptive blue water use to assess water impacts. However, for this study there was no information on where within a country the commodities were grown. Since water availability can vary considerably within a country, particularly large countries such as Mexico and the US, from which the vast majority of the Mexican diet originates, it was not possible to meaningfully apply water scarcity factors. Country-level factors could have been used, but considering the large uncertainties in country-level factors (especially for large countries such as Mexico and the US) we saw little value in using these.

Additionally, as the country-level AWARE factors for Mexico and the US are very similar, it would not have affected the results in this case. It should be stressed that the indicator for sustainability in relation to water use selected for this study does not capture the impact of water use, but it assesses the volume of blue water used to produce the diets. For a further discussion on benefits and drawbacks of different ways to assess water use, see e.g. (15).

Land use refers here to the total amount of agricultural land required for producing the Mexican diets, expressed in square meters. As agricultural land varies in productive capacity, different land use types should ideally be accounted for, most importantly land only suitable for pasture and land suitable for cropping (16). In this study, however, we used the total aggregated land use in order to simplify the analysis. This overestimates land use for diets high in ruminant products (16). The indicators used for land and water use in this study are both pressure indicators, i.e., they communicate the pressure human activities place on ecosystems rather than the potential impact due to such pressures (1).

The biodiversity loss associated with the use of land for producing the Mexican diets was assessed with the method developed by Chaudhary & Brooks (17), which accounts for regional species loss from land occupied by agriculture (reduction or removal of species that would otherwise exist on that land) by accounting for the relative abundance of those species within the region and the overall global threat level for the affected species. The method accounts for five broad land uses (managed forest, plantations, pasture, cropland, and urban), three land use intensities (minimal, light and intense), and five taxa (plants, mammals, birds, amphibians, and reptiles). There are still several limitations with this method, including poor resolution in land types and land management practices, and there are large gaps in current knowledge on how damaging a particular land use type is to different taxa in different parts of the world. The pathway from land use to biodiversity damage is highly complex, making it challenging to include all aspects in a single indicator (17).

Supplementary Figure 4. Methodological scheme for estimation of environmental footprint indicators of plant-based food using dietary data from SFFQ

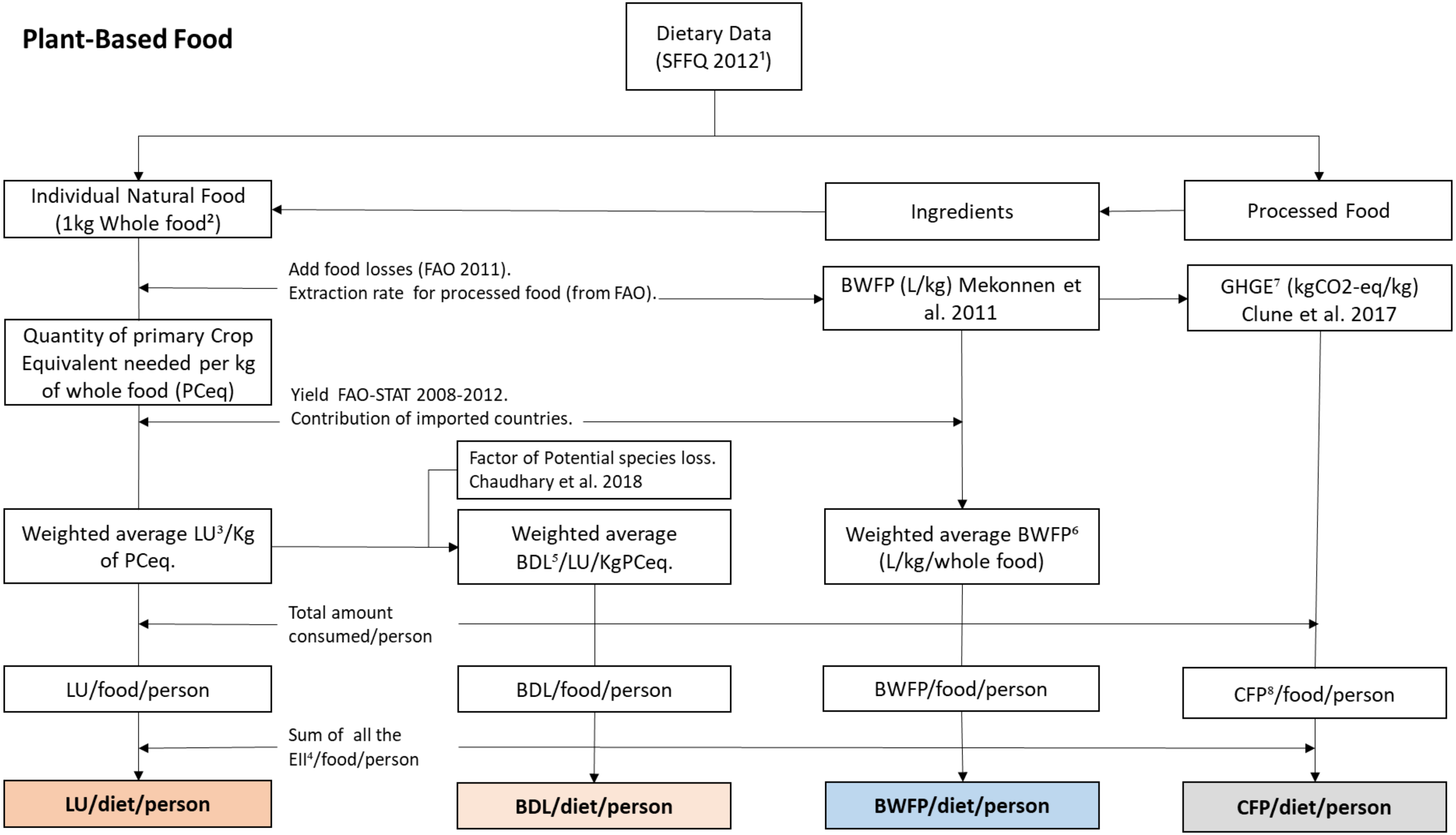

[1]Semi-quantitative food frequency questionnaire of the National Health and Nutrition Survey (ENSANUT-2012).
[2]Includes edible and non-edible portions (as purchased: with peel, seed, husk, etc.).
[3]Land use. [4]Environmental footprint per food consumed per person. [5]Biodiversity loss (potential number of species loss) (18). [6]Blue Water footprint (19). [7]Greenhouse gas emissions. [8]Carbon footprint (8).

Supplementary Figure 5 Methodological scheme for estimation of environmental footprint indicators of animal-based food using dietary data from SFFQ

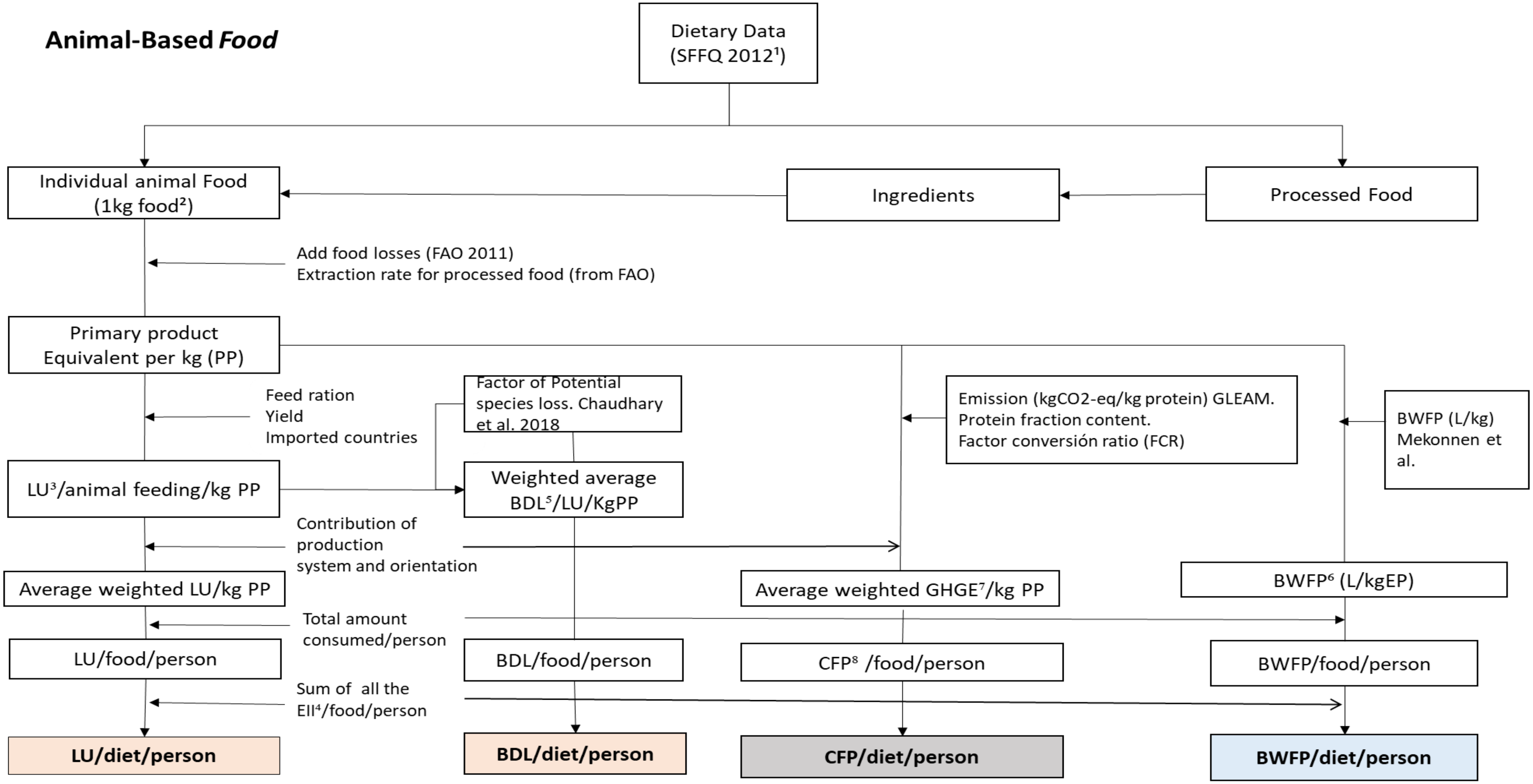

[1]Semi-quantitative food frequency questionnaire of the National Health and Nutrition Survey (ENSANUT-2012). [2]Includes edible and non-edible portions (as purchased: with peel, seed, husk, etc.) [3]Land use [4]Environmental footprint indicators per food consumed per person. [5]Biodiversity loss (potential number of species loss) (18). [6]Blue water footprint (19). [7]Greenhouse gas emissions. [8]Carbon footprint (8).

Supplementary Table Supplementary Table 2. Data used to calculate the environmental footprint indicators of plant-based food in SFFQ-ENSANUT 2012

N°	Food_name (SFFQ)	Country of Production & Importers	Contribution to Food Supply (Fraction)	Primary Production Data from FAOSTAT (2008-2013)		% Food losses of Edible Mass in the Food Supply (FAO-2011 Latin-America)			
				FAO-name	Yield (Ton/Ha)	Post-Harvest	Process	Distribution	House Consumption
1	MAIZ BLANCO	Mexico	1.00	Maize	3.18	0.04	0.02	0.04	0.10
3	MASA TRATADA CON CAL DE MAIZ BLANCO	Mexico	1.00	Maize	3.18	0.04	0.02	0.04	0.10
2	FRIJOL NEGRO	México	0.89	Beans, dry	0.73	0.03	0.08	0.02	0.02
3	FRIJOL NEGRO	USA (97.52%)	0.11	Beans dry	1.98	0.00	0.05	0.01	0.02
6	AJO	Mexico	1.00	Garlic	9.98	0.10	0.20	0.12	0.10
7	CHICHARO	Mexico	0.12	Beans, green	9.25	0.10	0.20	0.12	0.10
8	CHICHARO	USA (71.46)	0.63	Beans, green	4.68	0.04	0.02	0.12	0.10
9	CHICHARO	Guatemala (20.82	0.19	Beans, green	5.87	0.10	0.20	0.12	0.10
10	Chile poblano	Mexico	1.00	Chilies and	15.56	0.10	0.20	0.12	0.10
11	EJOTES	Mexico	1.00	String beans	9.78	0.03	0.08	0.02	0.02
12	Nopales	Mexico	1.00	Leaves of prickly pear[1]	64.64	0.10	0.20	0.12	0.10
15	Pepino	Mexico	1.00	Cucumbers and gherkins	34.50	0.10	0.20	0.12	0.10
16	ZANAHORIA	Mexico	1.00	Carrots and turnips	25.98	0.10	0.20	0.12	0.10
17	Fresa	Mexico	1.00	Strawberries	35.80	0.10	0.20	0.12	0.10
18	Jícama	Mexico	1.00	Jicama (no FAO)	27.69	0.10	0.20	0.12	0.10
19	Papaya	Mexico	1.00	Papaya	44.80	0.10	0.20	0.12	0.10
20	Piña	Mexico	1.00	Pineapples	42.85	0.10	0.20	0.12	0.10
21	PLATANO MACHO	Mexico	1.00	Bananas	28.72	0.10	0.20	0.12	0.10
22	Toronja	Mexico	1.00	Grapefruit *inc pomelos	23.62	0.10	0.20	0.12	0.10
23	Uvas	Mexico	1.00	Grapes	11.63	0.10	0.20	0.12	0.10
25	Palomitas de maíz	USA	0.97	Maize	9.29	0.04	0.02	0.04	0.10
26	Palomitas de maíz	MEXICO	0.03	Maize	3.18	0.04	0.02	0.04	0.10
28	MAIZ PRECOCIDO (PARA POZOLE)	Mexico	1.00	Maize	3.18	0.04	0.02	0.04	0.10
30	CHAMPIÑONES NATURALES	Mexico	1.00	Mushrooms and truffles	297.89	0.10	0.20	0.12	0.10
31	CHILE GUAJILLO (fresh)	Mexico	1.00	Chillies and peppers, green	15.56	0.10	0.20	0.12	0.10
32	Naranja o mandarina	Mexico	1.00	Orange	12.22	0.10	0.20	0.12	0.10
33	Manzana o pera	Mexico	0.77	Apples	9.35	0.10	0.20	0.12	0.10
34	Manzana o pera	USA (95.23%)	0.23	Apples	31.26	0.04	0.02	0.12	0.10
36	Melón o sandía	Mexico	1.00	Watermelons	24.77	0.10	0.20	0.12	0.10
37	Durazno/melocotón	Mexico	0.81	Peaches and nectarines	4.86	0.10	0.20	0.12	0.10
38	Durazno/melocotón	USA(83.75%)	0.15	Peaches and nectarines	20.50	0.04	0.02	0.12	0.10
39	Durazno/melocotón	CHILE (16.25%)	0.04	Peaches and nectarines	19.58	0.10	0.20	0.12	0.10
41	JITOMATE SALADET	Mexico	1.00	Tomatoes	30.01	0.10	0.20	0.12	0.10
42	Chayote	Mexico	1.00	Pumpkins, squash and gourds	16.79	0.10	0.20	0.12	0.10
43	Calabacita	Mexico	1.00	Pumpkins, squash and gourds	16.79	0.10	0.20	0.12	0.10

Supplementary Table 2 (Continued). Data used to calculate the environmental footprint indicators of plant-based food in SFFQ-ENSANUT 2012

N°	FAO-name	Primary Crop Equivalent/ kg whole	Primary Crop Equivalent/ kg whole	Non-Edible CF* (1/CF)	Factor from Edible to whole food	BDL Caracterization factor	Water footprint Blue ((L/kg))	Clune et al. GHGE (kgCO2e/kg)
1	Maize	1.23	1.16	1.00	1.00	5.40E-10	61.76	0.63
3	Maize	1.23	1.16	1.00	1.00	5.40E-10	61.76	0.63
2	Beans, dry	1.17	1.04	1.00	1.00	5.40E-10	174.26	0.62
3	Beans dry	1.08	1.03	1.00	1.00	7.00E-11	427.9	0.62
6	Garlic	1.75	1.26	0.97	1.03	5.40E-10	474.95	0.57
7	Beans, green	1.75	1.26	0.44	2.27	5.40E-10	70.58	0.51
8	Beans, green	1.34	1.26	0.44	2.27	5.40E-10	133.91	0.51
9	Beans, green	1.75	1.26	0.44	2.27	1.00E-09	2.06	0.51
10	Chilies and	1.75	1.26	0.80	1.25	5.40E-10	81.72	0.60
11	String beans	1.17	1.04	0.90	1.11	5.40E-10	79.19	0.51
12	Leaves of prickly pear[1]	1.75	1.26	0.78	1.28	5.40E-10	161.77	0.47
15	Cucumbers and gherkins	1.75	1.26	0.83	1.20	5.40E-10	31.21	0.33
16	Carrots and turnips	1.75	1.26	0.82	1.22	5.40E-10	49.28	0.22
17	Strawberries	1.75	1.26	0.96	1.04	5.40E-10	89.86	0.65
18	Jicama (no FAO)	1.75	1.26	0.93	1.08	5.40E-10	25.56	0.5
19	Papaya	1.75	1.26	0.68	1.47	5.40E-10	62.61	0.34
20	Pineapples	1.75	1.26	0.53	1.89	5.40E-10	23.46	0.72
21	Bananas	1.75	1.26	0.58	1.72	5.40E-10	180.66	0.79
22	Grapefruit *inc pomelos	1.75	1.26	0.66	1.52	5.40E-10	118.71	0.5
23	Grapes	1.75	1.26	0.68	1.47	5.40E-10	97	0.41
25	Maize	1.23	1.16	1.00	1.00	7.00E-11	63.29	0.63
26	Maize	1.23	1.16	1.00	1.00	5.40E-10	61.76	0.63
28	Maize	1.23	1.16	1.00	1.00	5.40E-10	61.76	0.63
30	Mushrooms and truffles	1.75	1.26	1.00	1.00	5.40E-10	161.77	0.27
31	Chillies and peppers, green	1.75	1.26	0.86	1.16	5.40E-10	81.72	0.6
32	Orange	1.75	1.26	0.71	1.41	5.40E-10	217.27	0.35
33	Apples	1.75	1.26	0.72	1.39	5.40E-10	400.38	0.36
34	Apples	1.34	1.26	0.72	1.39	7.00E-11	243.89	0.36
36	Watermelons	1.75	1.26	0.49	2.04	5.40E-10	60.12	0.32
37	Peaches and nectarines	1.75	1.26	0.88	1.14	5.40E-10	584.95	0.54
38	Peaches and nectarines	1.34	1.26	0.88	1.14	7.00E-11	492.12	0.54
39	Peaches and nectarines	1.75	1.26	0.88	1.14	1.30E-10	187.98	0.54
41	Tomatoes	1.75	1.26	0.88	1.14	5.40E-10	84.52	0.46
42	Pumpkins, squash and gourds	1.75	1.26	0.84	1.19	5.40E-10	161.77	0.33
43	Pumpkins, squash and gourds	1.75	1.26	0.71	1.41	5.40E-10	26.42	0.33

Supplementary Table 2 (Continued). Data used to calculate the environmental footprint indicators of plant-based food in SFFQ-ENSANUT 2012

N°	Food_name (SFFQ)	Country of Production & Importers	Contribution to Food Supply (Fraction)	Primary Production Data from FAOSTAT (2008-2013)		% Food losses of Edible Mass in the Food Supply (FAO-2011 Latin-America)			
				FAO-name	Yield (Ton/Ha)	Post-Harvest	Process	Distribution	House Consumption
44	Brócoli o coliflor	Mexico	1.00	Cabbages and other brassicas	32.75	0.10	0.20	0.12	0.10
45	ELOTE BLANCO	Mexico	1.00	Maize, green	11.39	0.10	0.20	0.12	0.10
46	Lechuga	Mexico	1.00	Lettuce and chicory	20.56	0.10	0.20	0.12	0.10
47	Broccoli, raw	Mexico	1.00	Cabbages and other brassicas	32.75	0.10	0.20	0.12	0.10
48	Carrots, raw or frozen	Mexico	1.00	Carrots and turnips	25.98	0.10	0.20	0.12	0.10
49	Peas, green, raw	Mexico	1.00	Beans, green	4.85	0.10	0.20	0.12	0.10
51	Chickpea	Mexico	1.00	Chick peas	1.70	0.03	0.08	0.02	0.02
52	Lentils	Mexico	1.00	Lentils	0.95	0.03	0.08	0.02	0.02
53	Lenteja, garbanzo, haba amarilla o alubia	Mexico	1.00	Grouped beans mean	1.32	0.03	0.08	0.02	0.02
54	Cacahuates (peanut)	Mexico	1.00	Peanut	1.67	0.05	0.09	0.10	0.04
55	Chiles frescos	Mexico	1.00	Chillies and peppers, green	15.56	0.10	0.20	0.12	0.10
56	Tomate verde y jitomate	Mexico	1.00	Tomatoes	30.01	0.10	0.20	0.12	0.10
57	Cebolla	Mexico	1.00	Onions, shallots, green	11.69	0.10	0.20	0.12	0.10
58	Plátano	Mexico	1.00	Bananas	28.72	0.10	0.20	0.12	0.10
59	Guayaba	Mexico	1.00	Mangoes, mangosteens, guavas	9.29	0.10	0.20	0.12	0.10
60	Mango	Mexico	1.00	Mangoes, mangosteens, guavas	9.29	0.10	0.20	0.12	0.10
61	Hojas Verdes (acelgas, espinacas, quelites)	Mexico	1.00	Spinach	11.98	0.10	0.20	0.12	0.10
62	Col	Mexico	1.00	Cabbages and other brassicas	32.75	0.10	0.20	0.12	0.10
63	AGUACATE MEAN	Mexico	1.00	Avocados	9.90	0.10	0.20	0.12	0.10
64	PAPA AMARILLA	Mexico	1.00	Potatoes	27.26	0.10	0.20	0.12	0.10
65	Limón	Mexico	1.00	Lemons and limes	14.02	0.10	0.20	0.12	0.10
66	Chile seco	Mexico	1.00	Chillies and peppers, dry	1.75	0.10	0.20	0.12	0.10
67	Peper ground (pimienta molida)	Mexico	1.00	Pepper *piper spp dry.	1.51	0.10	0.20	0.12	0.10
68	Soybean	Mexico	0.17	Soybeans	1.61	0.03	0.08	0.02	0.02
69	Soybean	USA	0.83	Soybeans	2.81	0.00	0.05	0.01	0.04
71	Tamarindo (to raw portion)	Mexico	1.00	Tamarindo "other tropical fruit"	4.46	0.10	0.20	0.12	0.10
72	Wheat	Mexico	1.00	Wheat	5.31	0.04	0.02	0.04	0.10

Supplementary Table 2 (Continued). Data used to calculate the environmental footprint indicators of plant-based food in SFFQ-ENSANUT 2012

N°	FAO-name	Primary Crop Equivalent/ kg whole	Primary Crop Equivalent/ kg whole	Non-Edible CF* (1/CF) (INSP)	Factor from Edible to whole food	BDL Caracterization factor	Water footprint	Clune et al.
							Blue ((L/kg))	GHGE (kgCO2e/kg)
44	Cabbages and other brassicas	1.75	1.26	0.69	1.45	5.40E-10	58.67	0.32
45	Maize, green	1.75	1.26	0.59	1.69	5.40E-10	90.19	0.63
46	Lettuce and chicory	1.75	1.26	0.75	1.33	5.40E-10	49.01	0.38
47	Cabbages and other brassicas	1.75	1.26	0.69	1.45	5.40E-10	58.67	0.32
48	Carrots and turnips	1.75	1.26	0.82	1.22	5.40E-10	49.28	0.22
49	Beans, green	1.75	1.26	0.44	2.27	5.40E-10	200.29	0.51
51	Chick peas	1.17	1.04	1.00	1.00	5.40E-10	47.79	0.67
52	Lentils	1.17	1.04	1.00	1.00	5.40E-10	267.59	1.03
53	Grouped beans mean	1.17	1.04	1.00	1.00	5.40E-10	139.38	0.85
54	Peanut	1.34	1.16	0.84	1.19	5.40E-10	2418.57	0.87
55	Chillies and peppers, green	1.75	1.26	0.86	1.16	5.40E-10	81.72	0.6
56	Tomatoes	1.75	1.26	0.89	1.12	5.40E-10	84.52	0.46
57	Onions, shallots, green	1.75	1.26	0.81	1.23	5.40E-10	87.29	0.18
58	Bananas	1.75	1.26	0.58	1.72	5.40E-10	180.66	0.79
59	Mangoes, mangosteens, guavas	1.75	1.26	0.83	1.20	5.40E-10	427.76	0.5
60	Mangoes, mangosteens, guavas	1.75	1.26	0.59	1.69	5.40E-10	427.76	0.5
61	Spinach	1.75	1.26	0.81	1.23	5.40E-10	97.68	0.54
62	Cabbages and other brassicas	1.75	1.26	0.80	1.25	5.40E-10	58.67	0.32
63	Avocados	1.75	1.26	0.53	1.89	5.40E-10	265.5	1.3
64	Potatoes	1.75	1.26	0.82	1.22	5.40E-10	112.02	0.2
65	Lemons and limes	1.75	1.26	0.64	1.56	5.40E-10	196.45	0.3
66	Chillies and peppers, dry	1.75	1.26	1.00	1.00	5.40E-10	2835.34	0.6
67	Pepper *piper spp dry.	1.75	1.26	1.00	1.00	5.40E-10	954.29	0.6
68	Soybeans	1.17	1.04	1.00	1.00	5.40E-10	767.93	0.58
69	Soybeans	1.11	1.05	1.00	1.00	5.40E-10	162.04	0.58
71	Tamarindo "other tropical fruit"	1.75	1.26	0.80	1.25	5.40E-10	406.07	0.5
72	Wheat	1.23	1.16	1.00	1.00	5.40E-10	622.21	0.51

Supplementary Table 2 (Continued). Data used to calculate the environmental footprint indicators of animal-based food in SFFQ-ENSANUT 2012

N°	FAO-name	Primary Crop Equivalent/ kg whole	Primary Crop Equivalent/ kg whole	Non-Edible CF* (1/CF) (INSP)	Factor from Edible to whole food	BDL Caracterization factor	Water footprint	Gleam
							Blue ((L/kg))	GHGE (kgCO2e/kg)
73	Eggs	1.00	1.00	0.88	1.14	1.10E-09	294	0.78
74	Bovine Meat	1.19	1.12	1.00	1.00	2.70E-08	801	56.12
75	Poultry Meat	1.19	1.12	1.00	1.00	2.30E-09	364	1.58
76	Poultry Meat	1.19	1.12	1.00	1.00	2.30E-09	364	1.58
77	Pigmeat	1.19	1.12	1.00	1.00	5.60E-09	837	7.13
78	Poultry Meat	1.19	1.12	1.00	1.00	2.30E-09	364	1.58
79	Eggs	1.00	1.00	0.88	1.14	1.10E-09	294	0.78
80	Lard	NE*	NE*	NE*	NE*	NE*	NE*	NE*
81	Fresh fish	1.34	1.16	0.55	1.82	ND	ND	4.41
82	Milk - Excluding Butter	1.23	1.13	1.00	1.00	9.80E-10	139	1.72
83	Milk - Excluding Butter	1.23	1.13	1.00	1.00	9.80E-10	139	1.72
84	Milk - Excluding Butter	1.23	1.13	1.00	1.00	9.80E-10	139	1.72
85	Dried fish (cod)	1.34	1.16	0.55	1.82	ND	ND	3.49
86	Shrimps	1.34	1.16	0.82	1.22	ND	ND	4.41
87	Milk - Excluding Butter	1.23	1.13	1.00	1.00	9.80E-10	139	1.72
88	Bovine Meat	1.19	1.12	1.00	1.00	2.70E-08	801.46	56.12

*Non-estimated
**ND. No data for water footprint in Mekonnen et al. (19), instead we used an estimate from Poor et al. (20).

Supplementary Table 3. Data used to calculate the environmental footprint indicators of processed animal and plant-based food in SFFQ-ENSANUT 2012

N°	Food_name (SFFQ)	Country of Production & Importers	Contribution to Food Supply (Fraction)	Primary Production Data from FAOSTAT (2008-2013)		% Food losses of Edible Mass in the Food Supply (FAO-2011 Latin-America)				Extraction rate (FAO)	Primary Crop Equivalent/kg whole food (PCeq) LU	Primary Crop Equivalent/kg whole food (PCeq) GHGE	BDL Caracterization factor (Intense LU)	Water footprint Mekonnen et al.				Clune et al.
				FAO-name	Yield (Ton/Ha)	Post-Harvest	Process	Distribution	House Consumption					Green (L/kg)	Blue (L/kg)	Grey (L/kg)	Total (L/kg)	GHGE (kgCO2e/kg)
1	ARROZ PULIDO (CRUDO)	Mexico	0.22	Rice, paddy	5.04	0.04	0.02	0.04	0.10	0.70	1.76	1.65	5.40E-10	1788	503	234	2525	2.66
2	ARROZ PULIDO (CRUDO)	USA	0.78	Rice, paddy	7.89	0.02	0.01	0.02	0.27	0.72	1.99	1.94	7.00E-11	610	1222	268	2099	2.66
3	Milled paddy rice for breakfast cereal	Mexico	0.22	Rice, paddy	5.04	0.04	0.02	0.04	0.10	0.64	1.93	1.82	5.40E-10	1946	548	255	2749	2.66
4	Milled paddy rice for breakfast cereal	USA	0.78	Rice, paddy	7.89	0.02	0.01	0.02	0.27	0.68	2.10	2.04	5.40E-10	664	1330	291	2285	2.66
5	HARINA DE MAIZ PARA ATOLE (MAICE	Mexico	1.00	Maize	3.18	0.04	0.02	0.04	0.10	0.86	1.43	1.35	5.40E-10	1899	63	366	2328	0.63
6	ACEITE DE CARTAMO (Cartamo oil)	México	0.80	Safflower seed	1.48	0.03	0.08	0.02	0.02	0.39	2.99	2.67	5.40E-10	7130	ND	91	7221	1.46
7	ACEITE DE CARTAMO (Cartamo oil)	USA	0.20	Safflower seed	1.52	0.00	0.05	0.01	0.04	0.41	2.70	2.57	7.00E-11	6339	230	775	7345	1.46
8	ACEITE DE MAIZ	Mexico	1.00	Maize	3.18	0.04	0.02	0.04	0.10	0.40	3.08	2.89	5.40E-10	3902	130	752	4784	0.63
9	PILONCILLO	Mexico	1.00	Sugar cane	70.83	0.04	0.02	0.04	0.10	0.09	13.37	12.58	5.40E-10	1147	282	124	1553	3.21
10	PASTAS SIN ENRIQUECER	Mexico	1.00	Wheat	5.31	0.04	0.02	0.04	0.10	0.69	1.78	1.68	5.40E-10	337	565	187	1089	0.51
11	Rapeseed *colza/canola	Mexico	0.75	Rapeseed	1.37	0.03	0.08	0.02	0.02	0.36	3.24	2.89	5.40E-10	6005		73	6078	1.46
12	Rapeseed *colza/canola	USA	0.25	Rapeseed	1.76	0.00	0.05	0.01	0.04	0.38	2.91	2.77	7.00E-11	5319	39	712	6070	1.46
13	Azucar	Mexico	1.00	Sugar cane	70.83	0.04	0.02	0.04	0.10	0.09	13.37	12.58	5.40E-10	1147	282	124	1553	3.21
14	Paletas y dulces de malvavisco (paleta	Mexico	1.00	Sugar cane	70.83	0.04	0.02	0.04	0.10	0.09	13.37	12.58	5.40E-10	1147	282	124	1553	3.21
15	Dulce (caramelos, paletas)	Mexico	1.00	Sugar cane	70.83	0.04	0.02	0.04	0.10	0.09	13.37	12.58	5.40E-10	1147	282	124	1553	3.21
16	Almond Unshelled to Shell almonds	Mexico	0.04	Almonds with shell	1.45	0.03	0.08	0.02	0.02	0.40	2.92	2.60	5.40E-10	3511	4190	370	8071	1.74
17	Almond Unshelled to Shell almonds	USA (69%)	0.69	Almonds, with shell	2.54	0.00	0.05	0.01	0.04	0.60	1.85	1.75	7.00E-11	2342	4075	6638	13055	1.74
18	Almond Unshelled to Shell almonds	CHILE (27%)	0.27	Almonds, with shell	2.80	0.03	0.08	0.02	0.02	0.35	3.33	2.97	1.30E-10	8865	3503	8197	20565	1.74
19	a) Té sin azúcar: Chamomile	Mexico	1.00	Chamomile	6.96	0.10	0.20	0.12	0.10	0.78	2.24	1.61	5.40E-10	ND	ND	ND	1047.92**	0.47
20	a) Café sin azúcar: Soluble coffe	Mexico	1.00	Coffee, green *	0.34	0.10	0.20	0.12	0.10	0.38	4.56	3.28	5.40E-10	27505	ND	816	28321	28.53
21	Sunflower oil	Mexico	0.75	Sunflower seed	1.47	0.03	0.08	0.02	0.02	0.39	2.99	2.67	5.40E-10	7130	ND	91	7221	3.6
22	Sunflower oil	ARGENTINA	0.18	Sunflower seed	1.72	0.03	0.08	0.02	0.02	0.40	2.92	2.60	3.60E-11	5391	23	52	5466	3.6
23	Sunflower oil	USA (24%)	0.06	Sunflower seed	1.64	0.00	0.05	0.01	0.04	0.41	2.70	2.57	7.00E-11	6339	230	775	7345	3.6
24	Palm oil	Mexico	0.62	Oil palm fruit	13.53	0.03	0.08	0.02	0.02	0.24	4.86	4.34	5.40E-10	3261	ND	412	3672	7.32
25	Palm oil	COSTA RICA	0.13	Oil palm fruit	17.01	0.03	0.08	0.02	0.02	0.24	4.86	4.34	2.60E-09	3399	ND	68	3467	7.32
26	Palm oil	GUATEMAL	0.12	Oil palm fruit	16.70	0.03	0.08	0.02	0.02	0.14	8.33	7.44	1.00E-09	2559	ND	58	2618	7.32
27	Palm oil	HONDURAS	0.12	Oil palm fruit	16.70	0.03	0.08	0.02	0.02	0.18	6.48	5.78	8.20E-10	3251	ND	120	3371	7.32
28	Soybean oil	México	0.17	Soybeans	1.61	0.03	0.08	0.02	0.02	0.16	7.29	6.51	5.40E-10	3129	438	ND	3567	6.32
29	Soybean oil	USA	0.83	Soybeans	2.81	0.00	0.05	0.01	0.04	0.19	5.83	5.54	7.00E-11	1560	92	10	1662	6.32

*The extraction ratio to estimate the equivalent primary crop of green coffee per kg of soluble coffee was based on the document conversions and statistics for world trade in coffee (http://www.intracen.org/guia-del-cafe/el-comercio-mundial-del-cafe/Conversiones-y-estadisticas/) (21).

**ND. No data for water footprint in Mekonnen et al. (18), instead we estimated WFP based on comparison of tea and coffee (21% x WFP of coffee).

Supplementary Table 3 (Continued). Data used to calculate the environmental footprint indicators of processed animal and plant-food in SFFQ-ENSANUT 2012

N°	Food_name (SFFQ)	Country of Production & Importers	Contribution to Food Supply (Fraction)	Primary Production Data from FAOSTAT (2008-2013)		% Food losses of Edible Mass in the Food Supply (FAO-2011 Latin-America)				Extraction rate (FAO)	Primary Crop Equivalent/kg whole food (PCeq) LU	Primary Crop Equivalent/kg whole food (PCeq) LU	BDL Caracterization factor (Intense LU)	Water footprint Mekonnen et al.				Clune et al.
				FAO-name	Yield (Ton/Ha)	Post-Harvest	Process	Distribution	House Consumption					Green (L/kg)	Blue (L/kg)	Grey (L/kg)	Total (L/kg)	GHGE (kgCO2e/kg)
30	Olive oil	España	0.77	Olives	2.54	0.01	0.05	0.01	0.04	0.21	5.33	5.01	6.90E-10	10809	2623	26	13458	5.42
32	Cacao paste	Mexico	1.00	Cocoa beans	0.59	0.10	0.20	0.12	0.10	0.80	2.19	1.58	5.40E-10	23618	ND	705	24323	8.00*
33	Cocoa Powered	Mexico	1.00	Cocoa beans	0.59	0.10	0.20	0.12	0.10	0.42	4.14	2.98	5.40E-10	15236	ND	455	15691	8.00*
34	Oats rolled	Mexico	1.00	Oats	1.54	0.04	0.02	0.04	0.10	0.58	2.12	2.00	5.40E-10	1610	2037	77	3724	0.44
35	Wheat (CEREAL FOR READY TO EAT CEREAL)	Mexico	1.00	Wheat	5.31	0.04	0.02	0.04	0.10	0.69	1.78	1.68	5.40E-10	371	622	206	1199	0.51
36	Wheat (BREAD)	Mexico	1.00	Wheat	5.31	0.04	0.02	0.04	0.10	0.69	1.78	1.61	5.40E-10	293	491	163	947	0.51
37	Leche sin lactosa descremada (Otra leche)	México	1.00	Milk EXC. Butter		0.06	0.02	0.08	0.04	1.00	1.23	1.13	9.80E-10	1586	134	117	1838	1.72
38	Leche semidescremada (Otra leche)	México	1.00	Milk EXC. Butter		0.06	0.02	0.08	0.04	1.00	1.23	1.13	9.80E-10	1639	139	121	1899	1.72
39	Leche descremada (Otra leche)	México	1.00	Milk EXC. Butter		0.06	0.02	0.08	0.04	1.00	1.23	1.13	9.80E-10	1586	134	117	1838	1.72
40	Leche liconsa (semi skim milk)	México	1.00	Milk EXC. Butter		0.06	0.02	0.08	0.04	1.00	1.23	1.13	9.80E-10	1639	139	121	1899	1.72
41	Leche evaporada descremada (Otra leche)	México	1.00	Milk EXC. Butter		0.06	0.02	0.08	0.04	0.32	3.84	3.54	9.80E-10	1586	134	117	1838	1.72
42	Leche evaporada entera (Otra leche)	México	1.00	Milk EXC. Butter		0.06	0.02	0.08	0.04	0.32	3.84	3.54	9.80E-10	1639	139	121	1899	1.72
43	Leche en polvo entera (Otra leche)	México	1.00	Milk EXC. Butter		0.06	0.02	0.08	0.04	0.12	10.24	9.44	9.80E-10	7377	625	545	8546	1.72
44	Leche en polvo descremada (Otra leche)	México	1.00	Milk EXC. Butter		0.06	0.02	0.08	0.04	0.12	10.24	9.44	9.80E-10	7377	625	545	8546	1.72
45	Leche en polvo entera	México	1.00	Milk EXC. Butter		0.06	0.02	0.08	0.04	0.12	10.24	9.44	9.80E-10	7377	625	545	8546	1.72
46	Queso panela o fresco o cottage	México	1.00	Milk EXC. Butter		0.06	0.02	0.08	0.04	0.10	12.29	11.32	9.80E-10	4941	418	365	5724	1.72
47	Quesos madurados (chihuahua, manchego, gou	México	1.00	Milk EXC. Butter		0.06	0.02	0.08	0.04	0.10	12.29	11.32	9.80E-10	7843	680	579	9101	1.72
48	Mantequilla	México	1.00	Butter		0.06	0.02	0.08	0.04	0.05	24.58	22.64	9.10E-10	2486	210	184	2880	11.52

* No data for carbon footprint in Clune et al. (8), instead we used an estimate from Ortiz-Rodríguez et al. (https://www.scielo.br/pdf/eagri/v36n2/1809-4430-eagri-36-2-0260.pdf) (22)

Supplementary Table 4. Feed ration composition (%) for meat and dairy by production system, based on GLEAM-i data for Mexico

**ORIENTATION**	**MEAT PRODUCTION**						**DAIRY PRODUCTION**					
**PRODUCTION SYSTEM**	**Grassland**			**Mixed**			**Grassland**			**Mixed**		
**Feed Ration components**	Adult Females	Meat animals (non feedlot)	Replacement animals and adult males	Adult Females	Meat animals (non feedlot)	Replacement animals and adult males	Adult Females	Meat animals (non feedlot)	Replacement animals and adult males	Adult Females	Meat animals (non feedlot)	Replacement animals and adult males
By-products from cottonseed	0.16	0.23	0.16	0.39	0.78	0.39	0.78	0.23	0.16	1.17	0.78	0.78
By-products from rape	0.13	0.2	0.13	0.33	0.66	0.33	0.66	0.2	0.13	0.99	0.66	0.66
By-products from soy	0.05	1.08	0.05	0.14	1.77	0.14	1.27	1.08	0.05	1.91	1.77	0.27
By-products from sugar beet							0	0	0	0	0	0
Crop residues from maize	5.42	4.81	5.42	5.26	4.15	5.26	4.43	4.81	5.42	3.87	4.15	4.98
Crop residues from millet							0	0	0	0	0	0
Crop residues from other	4.12	3.65	4.12	3.99	3.15	3.99	3.36	3.65	4.12	2.94	3.15	3.78
Crop residues from rice	10.44	9.27	10.44	10.12	7.99	10.12	8.53	9.27	10.44	7.46	7.99	9.59
Crop residues from sorghum	6.64	5.89	6.64	6.43	5.08	6.43	5.42	5.89	6.64	4.74	5.08	6.1
Crop residues from	9.72	8.63	9.72	9.42	7.44	9.42	7.93	8.63	9.72	6.94	7.44	8.93
Crop residues from wheat	11.46	10.18	11.46	11.11	8.77	11.11	9.36	10.18	11.46	8.19	8.77	10.53
Dry by-product from grain	0.14	1.71	0.14	0.35	2.94	0.35	2.19	1.71	0.14	3.29	2.94	0.69
Fodder beet	13.95	12.38	13.95	13.52	10.68	13.52	11.39	12.38	13.95	9.96	10.68	12.81
Fresh grass	1.95	1.73	1.95	1.89	1.49	1.89	1.59	1.73	1.95	1.39	1.49	1.79
Fresh mixture of grass and	5.9	5.24	5.9	5.72	4.52	5.72	4.82	5.24	5.9	4.21	4.52	5.42
Grains	0	5	0	0	7.5	0	5	5	0	7.5	7.5	0
Hay from adjacent areas												
Hay or silage from alfalfa	5.9	5.24	5.9	5.72	4.52	5.72	4.82	5.24	5.9	4.21	4.52	5.42
Hay or silage from cultivated	1.98	1.75	1.98	1.92	1.51	1.92	1.61	1.75	1.98	1.41	1.51	1.82
Hay or silage from grass and	1.98	1.75	1.98	1.92	1.51	1.92	1.61	1.75	1.98	1.41	1.51	1.82
Leaves from natural												
Maize	0	2	0	0	3	0	2	2	0	3	3	0
Maize gluten feed							0	0	0	0	0	0
Maize gluten meal	0	0.5	0	0	0.75	0	0.5	0.5	0	0.75	0.75	0
Molasses	1.13	1.7	1.13	2.83	5.67	2.83	5.67	1.7	1.13	8.5	5.67	5.67
Oil palm kernel expeller	0.39	0.58	0.39	0.96	1.93	0.96	1.93	0.58	0.39	2.89	1.93	1.93
Silage from whole grain	11.84	10.51	11.84	11.47	9.06	11.47	9.66	10.51	11.84	8.45	9.06	10.87
Silage from whole maize	6.71	5.96	6.71	6.51	5.14	6.51	5.48	5.96	6.71	4.79	5.14	6.16
Wet by-product from grain	0	0	0				0	0	0	0	0	0

Source: GLEAM-interactive tool for México (23).

Supplementary Table 5. Average feed ratio composition (%) for meat and dairy by production system, based on GLEAM-i data for Mexico

Cattle production	Meat Production		Milk Production	
	Grassland	Mixed	Grassland	Mixed
**Feed component (% of total DM feed intake)**				
**Crop for grains:**	2.33	3.50	4.67	7.00
Grains	1.67	2.50	3.33	5.00
Maize	0.67	1.00	1.33	2.00
**Total forrage - pastures**	48.33	45.26	45.25	40.12
Fodder beet	13.43	12.57	12.57	11.15
Fresh grass	1.88	1.76	1.76	1.56
Fresh mixture of grass and legumes	5.68	5.32	5.32	4.72
Hay or silage from alfalfa	5.68	5.32	5.32	4.72
Hay or silage from cultivated grass	1.90	1.78	1.78	1.58
Hay or silage from grass and legumes	1.90	1.78	1.78	1.58
Silage from whole grain plants.	11.40	10.67	10.67	9.46
Silage from whole maize plant	6.46	6.05	6.05	5.36
**Total by-products**	3.33	8.17	7.00	14.67
By-products from cottonseed	0.18	0.52	0.39	0.91
By-products from rape (canola)	0.15	0.44	0.33	0.77
By-products from soy	0.39	0.68	0.80	1.32
Dry by-product from grain industries	0.66	1.21	1.35	2.31
Maize gluten meal	0.17	0.25	0.33	0.50
Molasses	1.32	3.78	2.83	6.61
Oil palm kernel expeller	0.45	1.28	0.97	2.25
**Total crop residues**	46.01	43.08	43.09	38.21
Crop residues from maize	5.22	4.89	4.89	4.33
Crop residues from other grains	3.96	3.71	3.71	3.29
Crop residues from rice	10.05	9.41	9.41	8.35
Crop residues from sorghum	6.39	5.98	5.98	5.31
Crop residues from sugarcane	9.36	8.76	8.76	7.77
Crop residues from wheat	11.03	10.33	10.33	9.16
**TOTAL**	**100**	**100**	**100**	**100**

Source: GLEAM-interactive tool for México (23).

Supplementary Table 6. Feed ratio composition (%) for chicken and pig by production system, based on GLEAM-i data for Mexico

	Chicken			Pig		
**Production systems**	Meat Broiler	Egg Backyard	Egg Layers	Backyard	Intermediate	Industrial
**Grains:**	**71**	**33.08**	**72**	**41.3**	**58.45**	**64**
Grains from barley (Hordeum vulgare)	0	4.03	0	5.07	8.66	10
Grains from maize (Zea mays)	71	5.36	7	6.88	16.21	20
Grains from rice	0	8.58	0	10.38	3.54	0
Grains from sorghum	0	6.4	65	7.74	2.64	0
Grains from wheat (Triticum aestivum).	0	8.71	0	11.23	27.4	34
**Legumes:**	**0**	**3.65**	**0**	**4.42**	**1.5**	**0**
Beans from soy	0	2.26	0	**2.74**	**0.93**	**0**
Leguminous beans	0	1.39	0	1.68	0.57	0
**Forrages and fruit:**	**0**	**8.93**	**0**	**1.99**	**0.68**	**0**
Fruit from banana trees	0	1.64	0	1.99	0.68	0
Fresh grass	0	7.29	0			
**Total by-products**	**27**	**17.47**	**20**	**21.75**	**28.21**	**30**
By-product from oil production other than soy, cottonseed or palm oil.	0	2.9	10	**3.71**	**8.2**	**10**
By-products from cottonseed	0	6.51	0	**7.88**	**2.69**	**0**
By-products from soy (cake)	27	2.26	10	**3.04**	**11.43**	**15**
Dry by-product from grain industries	0	5.8	0	**7.12**	**5.89**	**5**
- Maiz yellow (bran)						
- Wheat (bran)						
**Total crop residues and household waste**	**0**	**36.86**	**0**	**30.41**	**6.96**	**0**
Crop residue from leguminous plants cultivation	0	3.21	0	**3.89**	**1.33**	**0**
Crop residues from sugarcane	0	12.01	0	**14.53**	**4.95**	**0**
Household food waste and other organic material used as feed	0	20	0	10		
Residual plant material such as stems from banana	0	1.64	0	1.99	0.68	0
**Excluded diet components**	**2**	**0**	**8**	**0.12**	**4.2**	**6**
Limestone		0				
SYNTHETIC		0		0.04	1.4	2
FISHMEAL				0.08	2.8	4
Total	100.00	99.99	100.00	99.99	100.00	100.00

Source: GLEAM-interactive tool for México (23).

Supplementary Table 7. Parameters for cattle production used in estimation of environmental footprint indicators by orientation and production system, based on GLEAM-i data for Mexico

Parameters for Cattle production (GLEAM 2010)	Unit	Meat Orientation		Dairy Orientation	
		Grassland System	Mixed System	Grassland System	Mixed System
Total feed intake	(kgDM/year)	34,488,198,625.02	23,074,303,460.37	12,772,826,708.50	9,003,257,436.32
System meat production in carcass weight	kg/year	792,165,541.81	526,494,592.62	264,069,383.88	204,906,475.20
Bone-free-meat (BFM) to carcass weight ratio	Fraction	0.7500	0.7500	0.7500	0.7500
Meat protein content	Fraction	0.2113	0.2113	0.2113	0.2113
Milk protein content	Fraction			0.0310	0.0310
Bone-free-meat (0.75 x total carcass weight)	kg/year	594,124,156.36	394,870,944.47	198,052,037.91	153,679,856.40
Milk production (Adult Females)	kg/year			5,827,381,922.63	4,648,752,164.26
Total meat protein (0.21 * Bone-free meat)	kg/year	125538434.2	83436230.57	41848395.61	32472553.66
Total milk protein (0.031 * Milk production)	kg/year	0	0	180,648,839.60	144,111,317.09
Total protein produce (Meat + milk protein)	kg/year	125538434.2	83436230.57	222497235.2	176583870.7
Meat protein contribution to total protein production (Allocation factor for meat in dairy production orientation)	Fraction			0.1881	0.1839
Milk protein (Allocation factor for milk in dairy production orientation)	Fraction			0.8119	0.8161
Meat contribution to the total production of meat (BFM)	Fraction	0.4431	0.2945	0.1477	0.1146
Milk contribution to the total Milk production	Fraction			0.5563	0.4437

Source: GLEAM-interactive tool for México (23)..

Supplementary Table 8. Parameters for chicken and pig production used in estimation of environmental footprint indicators by production system, based on GLEAM-i data for Mexico

Parameters for Chicken Production (GLEAM 2010)	Unit	Meat Broiler	Egg Backyard	Egg Layers
Total feed intake	kgDM/year	9,316,053,949.10	1,013,767,270.07	7,079,026,038.41
System meat production in carcass weight	kg/year	3,133,227,104.65	9,038,455.54	198,063,643.67
Bone-free-meat to carcass weight ratio	Fraction	0.7500	0.7500	0.7500
Meat protein content	Fraction	0.1900	0.1900	0.1900
Egg protein content	Fraction		0.1240	0.1240
Bone-free-meat (0.75 x total carcass weight)	kg/year	2,349,920,328.49	6,778,841.66	148,547,732.75
Eggs production of a given system (kg)	kg/year		2,914,439,481.87	2,914,439,481.87
Total meat protein (0.19 * Bone-free meat)	kg/year		1,287,979.91	28,224,069.22
Total egg protein (0.12 * Egg production)	kg/year		361,390,495.75	361,390,495.75
Total protein produce (Meat + Egg protein)	kg/year		362,678,475.67	389,614,564.97
Meat protein contribution to total protein produced (Allocation factor for meat for Egg production orientation)	Fraction		0.0036	0.0724
Egg protein contribution to the total protein produced (Allocation factor for Eggs for Egg production orientation)	Fraction		0.9964	0.9276
Meat contribution to the total production of meat (BFM)	Fraction	0.0524	0.0002	0.0033
Egg contribution to the total production of eggs	Fraction		0.2782	0.2782

Parameters for Pig Production (GLEAM 2010)	Unit	Backyard	Intermediate	Industrial
Total feed intake	kgDM/year	4,126,549,934.11	1,828,261,772.36	3,261,194,464.60
System meat production in carcass weight	kg/year	579,592,272.17	396,872,936.33	801,330,564.13
Bone-free-meat to carcass weight ratio	Fraction	0.650	0.650	0.650
Meat protein content	Fraction	0.202	0.202	0.202
Bone-free-meat (0.65 * total carcass weight)	kg/year	376,734,976.91	257,967,408.61	520,864,866.68
Total meat protein (0.20 * Bone-free meat)	kg/year	76,100,465.34	52,109,416.54	105,214,703.07
Meat contribution to the total production of meat (BFM)	Fraction	167,437,767.52	114,652,181.61	231,495,496.30

Source: GLEAM-interactive tool for México (23)

Supplementary Table 9. Average land use and average potential species loss per kg dry matter (DM) of each animal feed ration component

N	Feed ration components	Name Primary Crops (FAO)	DM(%) of primary crops (Gleam)[1]	Average_LU/kgDM feed ration	Average Potential species loss per kg of DM product (Intense)	Average Potential species loss per kg of DM product (Light)
1	Beans from soy	Soybean	0.91	3.60	7.10E-10	6.92E-10
2	By-product from oil production	Rapessed	0.92	6.30	7.21E-10	7.02E-10
3	By-products from cottonseed	Seed Cotton	0.94	3.06	1.25E-09	1.21E-09
4	By-products from soy (cake)	Soybean	0.91	3.60	7.10E-10	6.92E-10
5	Maiz yellow (bran)	Yellow maiz	0.87	1.76	5.32E-10	5.18E-10
6	Wheat (bran)	Wheat	0.87	2.17	1.17E-09	1.14E-09
7	Fresh grass	Rye grass en verde	0.90	0.29	1.55E-10	1.35E-10
8	Fruit from banana trees	Banana	0.20	1.74	9.44E-10	9.19E-10
9	Grains from barley (Hordeum	Barley	0.89	4.39	6.09E-11	5.95E-11
10	Grains from maize (Zea mays)	Maize yellow	0.87	1.76	5.32E-10	5.18E-10
11	Grains from rice	Rice	0.89	1.60	3.39E-10	3.30E-10
12	Grains from sorghum	Sorghum	0.89	3.00	1.07E-09	1.04E-09
13	Grains from wheat (Triticum	Wheat	0.89	2.17	1.17E-09	1.14E-09
15	Leguminous beans	Beans, dry	1.00	13.71	7.44E-09	7.23E-09
16	Maize White grain	White maize	0.87	1.76	1.96E-09	1.91E-09
17	Fodder beet	Fodder beet **	0.25	2.31	1.25E-09	1.22E-09
18	Alfalfa green	Alfalfa green *	1.00	2.38	8.94E-10	7.82E-10
19	Sorghum forrage, green	Sorghum forrage, green *	1.00	2.22	6.70E-10	5.86E-10
20	Oat forrage	Oat forrage *	1.00	3.17	1.19E-09	1.04E-09
21	Silage from whole maize plant	Forrage maize (green) *	1.00	1.49	5.65E-10	5.50E-10
22	Maize gluten meal	Maize yellow	0.87	1.76	5.32E-10	5.18E-10
23	Molasses	Sugar can	0.32	0.44	2.39E-10	2.33E-10
24	Oil palm kernel expeller	Oil palm fruit	0.53	1.27	1.08E-09	1.05E-09

Source: Yield of crops taken from FAOSTAT as the average yield of 2008-2012 (24). *Data on DM yield of alfalfa, sorghum, oat, and maize forage taken from Amendola et al. (25). DM factors taken from GLEAM (26) . **The DM factor of fodder beet corresponds to that of sugar beet.

Supplementary Table 10. Land use, biodiversity loss, and parameters used to estimate the carbon footprint of animal-based food in SFFQ.

Specie: Cattle (System-Orientation)	Unit	% Contribution system meat/milk	Meat/Milk emission intensity/kgProt	Contein of Prot/1kg product	Emission/kg Product	FCR	Lan use m2/kg product	Biodiversity Loss/kg product (INTENSE)	Biodiversity Loss/kg product (light)
LU Grass-Meat	kgCO2-eq/kgProt	0.44	338.48	0.21	71.08	58.05	63.00	2.59E-08	2.41E-08
LU Mix- Meat	kgCO2-eq/kgProt	0.29	321.07	0.21	67.42	58.44	66.06	2.68E-08	2.50E-08
LU Grass-Dairy-Meat	kgCO2-eq/kgProt	0.15	84.77	0.21	17.80	64.49	74.38	2.98E-08	2.78E-08
LU Mix-Dairy-Meat	kgCO2-eq/kgProt	0.11	88.54	0.21	18.59	58.58	72.41	2.83E-08	2.66E-08
LU Grass-Dairy-Milk	kgCO2-eq/kgProt	0.56	59.3	0.031	1.84	2.19	2.53	1.01E-09	9.45E-10
LU Mix-Dairy-Milk	kgCO2-eq/kgProt	0.44	50.46	0.031	1.56	1.94	0.00	9.36E-10	8.79E-10
Average emissions per meat beef	kgCO2-eq/kgProt	1.00	267.23	0.21	56.12		66.66	2.70E-08	2.52E-08
Average emissions per milk beef	kgCO2-eq/kgProt	1.00	55.38	0.031	1.72		1.41	9.79E-10	9.16E-10

Specie: Chicken (System-Orientation)	Unit	% Contribution system meat/egg	Meat/Egg emission intensity/kgProt	Contein of Prot/1kg product	Emission/kg Product	FCR	Lan use m2/kg product	Biodiversity Loss/kg product (Intense)	Biodiversity Loss/kg product (Light)
LU BROILER-Meat	kgCO2-eq/kgProt	0.94	7.88	0.19	1.50	3.96	8.80	2.26E-09	2.20E-09
LU BACKYARD- Meat	kgCO2-eq/kgProt	0.00	9.47	0.19	1.80	0.53	0.90	2.89E-10	2.81E-10
LU LAYER-Meat	kgCO2-eq/kgProt	0.06	14.84	0.19	2.82	3.45	10.58	3.02E-09	2.94E-09
LU BACKYARD-Egg	kgCO2-eq/kgProt	0.50	5.34	0.124	0.66	0.35	0.59	1.89E-10	1.83E-10
LU LAYER-Egg	kgCO2-eq/kgProt	0.50	7.16	0.124	0.89	2.25	6.91	1.97E-09	1.92E-09
Average emissions per chicken meat	kgCO2-eq/kgProt	1.00	8.30	0.19	1.58		8.89	2.30E-09	2.24E-09
Average emissions per chicken egg	kgCO2-eq/kgProt	1.00	6.25	0.124	0.78		3.75	1.08E-09	1.05E-09

Specie: Pig (System-Orientation)	Unit	% Contribution system meat/milk	Meat emission intensity/kgProt	Contein of Prot/1kg product	Emission/kg Product	FCR	Lan use m2/kg product	Biodiversity Loss/kg product (Intense)	Biodiversity Loss/kg product (light)
Backyard-Meat	kgCO2-eq/kgProt	0.33	36.76	0.202	7.43	10.95	22.71	7.23E-09	7.04E-09
Intermediate - Meat	kgCO2-eq/kgProt	0.22	36.78	0.202	7.43	7.09	18.74	5.19E-09	5.06E-09
Industrial - Meat	kgCO2-eq/kgProt	0.45	33.5	0.202	6.77	6.26	17.50	4.59E-09	4.47E-09
Average emissions per pig meat	kgCO2-eq/kgProt	1.00	35.30	0.202	7.13		19.48	5.59E-09	5.44E-09

Source: Calculation and parameters based on GLEAM-i emissions for Mexico.

Supplementary Table 11. Spearman correlation coefficients between diet quality (HEI-2015) and different environmental footprint indicators

	HEI-2015 score	
	Spearman coefficient (rho)	p-value
Land use, m^2/year/2000 kcal	-0.0198	0.328
Carbon footprint, kgCO_2eq/2000 kcal	-0.0945*	0.000
Blue water footprint, L/2000 kcal	0.2968*	0.000
Potential species loss per/2000 kcal x 10^{-10} *	-0.1395*	0.000

References:

1. Vanham D, Leip A, Galli A, Kastner T, Bruckner M, Uwizeye A, van Dijk K, Ercin E, Dalin C, Brandão M, et al. Environmental footprint family to address local to planetary sustainability and deliver on the SDGs. Sci Total Environ. Elsevier; 2019;693:133642.
2. Röös E. Analysing the Carbon Footprint of Food Insights for Consumer Communication. 2013;
3. Balcombe P, Speirs JF, Brandon NP, Hawkes AD. Methane emissions: choosing the right climate metric and time horizon. Environ Sci Process Impacts. Royal Society of Chemistry; 2018;20:1323–39.
4. Myhre G, ShindellMyhre G, Shindell D, Bréon F-M, Collins W, Fuglestvedt J, Huang J, Koch D, Lamarque J-F, Lee D, Mendoza B, Nakajima T, Robock A, Stephens G, Takemura T, Zhang H D, Bréon F-M, Collins W, Fuglestvedt J, Huang J, Koch D, Lamarque J-F, Lee D, ZH. Anthropogenic and natural radiative forcing. In: Stocker TF, Qin D, Plattner G-K, Tignor M, Allen SK, Boschung J, Nauels A, Xia Y, Bex V MP, editor. Climate change 2013: the physical science basis Contribution of working group I to the fifth assessment report of the intergovernmental panel on climate change. Cambridge: Cambridge Univ. Press; 2013.
5. Lynch J, Cain M, Frame D, Pierrehumbert R. Agriculture's Contribution to Climate Change and Role in Mitigation Is Distinct From Predominantly Fossil CO2-Emitting Sectors. Front Sustain Food Syst. Frontiers Media S.A.; 2021;4:300.
6. Rogelj J, Schleussner CF. Unintentional unfairness when applying new greenhouse gas emissions metrics at country level. Environ Res Lett. IOP Publishing; 2019;14:114039.
7. Steinfeld H, Gerber P, Wassenaar T, Castel V, Rosales M, Haan C. Livestock´s long shadow. 2006.
8. Clune S, Crossin E, Verghese K. Systematic review of greenhouse gas emissions for different fresh food categories. J Clean Prod. 2017;140:766–83.
9. Forster, P.; Ramaswamy, V.; Artaxo, P.; Berntsen T. et al. Changes in Atmospheric Constituents and Radiative Forcing" Climate Change 2013: The Physical Science Basis. Contribution of Working Group I to the Fourth Assessment Report of the Intergovernmental Panel on Climate Change. 2017 p. 129–234.
10. Hoekstra AY, Chapagain AK, Mekonnen MM, Aldaya MM. The water footprint assessment manual: Setting the global standard. Routledge; 2011.
11. Pfister S, Boulay AM, Berger M, Hadjikakou M, Motoshita M, Hess T, Ridoutt B, Weinzettel J, Scherer L, Döll P, et al. Understanding the LCA and ISO water footprint: A response to Hoekstra (2016) "A critique on the water-scarcity weighted water footprint in LCA." Ecol Indic. Elsevier; 2017;72:352–9.
12. Kounina A, Margni M, Bayart JB, Boulay AM, Berger M, Bulle C, Frischknecht R, Koehler A, Milà I Canals L, Motoshita M, et al. Review of methods addressing freshwater use in life cycle inventory and impact assessment. Int J Life Cycle Assess. Springer Verlag; 2013;18:707–21.
13. Quinteiro P, Ridoutt BG, Arroja L, Dias AC. Identification of methodological challenges remaining in the assessment of a water scarcity footprint: a review. Int J Life Cycle Assess 2017 231. Springer; 2017;23:164–80.
14. Boulay AM, Bare J, Benini L, Berger M, Lathuillière MJ, Manzardo A, Margni M, Motoshita M, Núñez M, Pastor AV, et al. The WULCA consensus characterization model for water scarcity footprints: assessing impacts of water consumption based on available water remaining (AWARE). Int J Life Cycle Assess. Springer Verlag; 2018;23:368–78.
15. Ran Y, van Middelaar CE, Lannerstad M, Herrero M, de Boer IJM. Freshwater use in livestock production—To be used for food crops or livestock feed? Agric Syst. Elsevier; 2017;155:1–8.
16. Ridoutt BG, Hendrie GA, Noakes M. Dietary Strategies to Reduce Environmental Impact: A Critical Review of the Evidence Base. Adv Nutr. Oxford University Press; 2017;8:933.
17. Curran M, De Souza DM, Antón A, Teixeira RFM, Michelsen O, Vidal-Legaz B, Sala S, Milà I Canals L. How Well Does LCA Model Land Use Impacts on Biodiversity? - A Comparison with

Approaches from Ecology and Conservation. Environ Sci Technol. American Chemical Society; 2016;50:2782–95.
18. Chaudhary A, Brooks TM. Land use intensity-specific global characterization factors to assess biodiversity footprints. Environ Sci Technol. 2018;52:5094–104.
19. Mekonnen MM, Hoekstra AY. Hydrology and Earth System Sciences The green, blue and grey water footprint of crops and derived crop products. Hydrol Earth Syst Sci. 2011;15:1577–600.
20. Poore J, Nemecek T. Reducing food's environmental impacts through producers and consumers. Science (80-). American Association for the Advancement of Science; 2018;360:987–92.
21. El comercio mundial del café-Conversiones y estadísticas.
22. Ortiz-Rodríguez OO, Villamizar-Gallardo RA, Naranjo-Merino CA, García-Caceres RG, Castañeda-Galvís MT. on-line) Recebido pelo Conselho Editorial em: 15-10-2015 Aprovado pelo Conselho Editorial em: 2-2-2016 Eng. J Brazilian Assoc Agric Eng. 36:260–70.
23. FAO. Gleam-i [Internet]. Available from: https://gleami.apps.fao.org/
24. FAO. FAOSTAT [Internet]. Crops. 2019. Available from: https://www.fao.org/faostat/en/
25. (PDF) Recursos forrajeros en México y sus perspectivas para la intensificación.
26. FAO. Global livestock environmental assessment model. Version 2. Rome, Italy;
